# Genome-wide analyses of cell-free DNA for therapeutic monitoring of patients with pancreatic cancer

**DOI:** 10.1126/sciadv.ads5002

**Published:** 2025-05-21

**Authors:** Carolyn Hruban, Daniel C. Bruhm, Inna M. Chen, Shashikant Koul, Akshaya V. Annapragada, Nicholas A. Vulpescu, Sarah Short, Susann Theile, Kavya Boyapati, Bahar Alipanahi, Zachary L. Skidmore, Alessandro Leal, Stephen Cristiano, Vilmos Adleff, Julia S. Johannsen, Robert B. Scharpf, Zachariah H. Foda, Jillian Phallen, Victor E. Velculescu

**Affiliations:** ^1^ The Sidney Kimmel Comprehensive Cancer Center, Johns Hopkins University School of Medicine, Baltimore, MD, USA.; ^2^ Department of Oncology, Copenhagen University Hospital–Herlev and Gentofte, Herlev, Denmark.; ^3^ Delfi Diagnostics Inc., Baltimore, MD, USA.; ^4^ Department of Medicine, Copenhagen University Hospital–Herlev and Gentofte, Herlev, Denmark.; ^5^ Department of Clinical Medicine, Faculty of Health and Medical Sciences, University of Copenhagen, Copenhagen, Denmark.; ^6^ Department of Medicine, Johns Hopkins University School of Medicine, Baltimore, MD, USA.

## Abstract

Determining response to therapy for patients with pancreatic cancer can be challenging. We evaluated methods for assessing therapeutic response using cell-free DNA (cfDNA) in plasma from patients with metastatic pancreatic cancer in the CheckPAC trial (NCT02866383). Patients were evaluated before and after initiation of therapy using tumor-informed plasma whole-genome sequencing (WGMAF) and tumor-independent genome-wide cfDNA fragmentation profiles and repeat landscapes (ARTEMIS-DELFI). Using WGMAF, molecular responders had a median overall survival (OS) of 319 days compared to 126 days for nonresponders [hazard ratio (HR) = 0.29, 95% confidence interval (CI) = 0.11–0.79, *P* = 0.011]. For ARTEMIS-DELFI, patients with low scores after therapy initiation had longer median OS than patients with high scores (233 versus 172 days, HR = 0.12, 95% CI = 0.046–0.31, *P* < 0.0001). We validated ARTEMIS-DELFI in patients with pancreatic cancer in the PACTO trial (NCT02767557). These analyses suggest that noninvasive mutation and fragmentation-based cfDNA approaches can identify therapeutic response of individuals with pancreatic cancer.

## Teaser

Noninvasive mutation-based and fragmentation-based cfDNA approaches can identify response to therapy in pancreatic cancer.

## INTRODUCTION

In 2020, more than 450,000 people are estimated to have died from pancreatic cancer worldwide (*1*). Most pancreatic cancers are ductal adenocarcinomas (PDAC), and patients with PDAC have an extremely poor prognosis, especially when their disease is diagnosed at an advanced stage (*2*), with a grim 12% survival rate (*3*). For patients with metastatic or locally advanced nonresectable PDAC, the standard-of-care treatment is either chemotherapy or symptomatic management (*4*). Recent studies, however, suggest that some patients with advanced-stage disease respond to targeted therapies based on somatic mutations in their cancers or to immune checkpoint blockade treatment (*5*–*9*). Unfortunately, determining response early during therapy using currently available imaging techniques is imperfect, especially in small, isoattenuating lesions (*10*). Continuing ineffective therapy can be detrimental as tumors can double in size within 5 months in the absence of therapeutic response (*11*–*13*). Consequently, there is a need to develop noninvasive approaches that can provide a real-time and accurate assessment of response to therapy for patients with PDAC.

Current tools for monitoring PDAC include radiographic Response Evaluation Criteria in Solid Tumors (RECIST 1.1) assessment using computed tomography (CT) and evaluation of serum CA19-9 (*14*). However, each of these methods has shortcomings. CT scans can be misleading in patients treated with immunotherapy as immune infiltration can mask therapeutic response or appear as pseudo-progression in radiographs (*15*). In addition, not all patients have tumors that secrete CA19-9, such as patients who are Lewis antigen Le(a−b−), limiting the use of this protein as a biomarker for response to therapy (*16*).

Liquid biopsy approaches based on detection of cell-free DNA (cfDNA) have been developed for both detecting cancer and for monitoring patients with cancer (*17*). Mutation-based assays for liquid biopsy including targeted sequencing and droplet digital polymerase chain reaction (PCR) have both been used for longitudinal monitoring of patients with pancreatic cancer (*6*, *18*). These approaches rely on either known mutations from sequencing the patient-matched tumor or mutations prevalent in the tumor type, such as those at positions of hotspot alterations in *KRAS*. However, not all cancers harbor the hotspot mutations or have an available tumor biopsy that can be assessed by these approaches. In addition, sequence alterations due to white blood cell clonal hematopoesis can confound the ability to determine whether the mutation is tumor derived.

Whole-genome sequencing (WGS) of cfDNA has been used to identify rearrangements for sensitive cancer detection and monitoring during therapy (*19*). In addition, genome-wide analyses of cfDNA copy number changes have been used in applications for both detection and monitoring (*20*, *21*). Genome-wide analyses of sequence alterations have also been used for disease monitoring when combined with a priori tumor sequencing (*22*). cfDNA fragmentome approaches such as DNA evaluation of fragments for early interception (DELFI), incorporate cfDNA fragment characteristics from across the genome and have shown promise for early cancer detection (*23*–*26*). The DELFI approach uses low-coverage WGS and does not require a priori information of the somatic mutations in the patient’s cancer (*27*). Recent studies have demonstrated the utility of fragmentome-based approaches for monitoring disease during therapy by predicting mutant allele fractions (MAFs) and tumor burden through a tumor- and mutation-independent approach (*28*–*31*). Repeat element landscapes from cfDNA [analysis of repeat elements in disease (ARTEMIS)] have also recently been shown to have utility for detecting and monitoring cancer (*32*).

In this study, we developed and evaluated a tumor-informed [whole-genome MAF (WGMAF)] and a tumor-independent cfDNA approach (ARTEMIS-DELFI) in serial plasma samples from patients in a clinical trial who were treated with late-line immunotherapy combined with radiation therapy for metastatic pancreatic cancer. The results were validated in a separate clinical trial of patients with locally advanced or metastatic pancreatic cancer treated with first-line chemotherapy and were compared to other monitoring modalities, including molecular and imaging results as well as patient outcome.

## RESULTS

### Patient population and approach

**Fig. 1.**
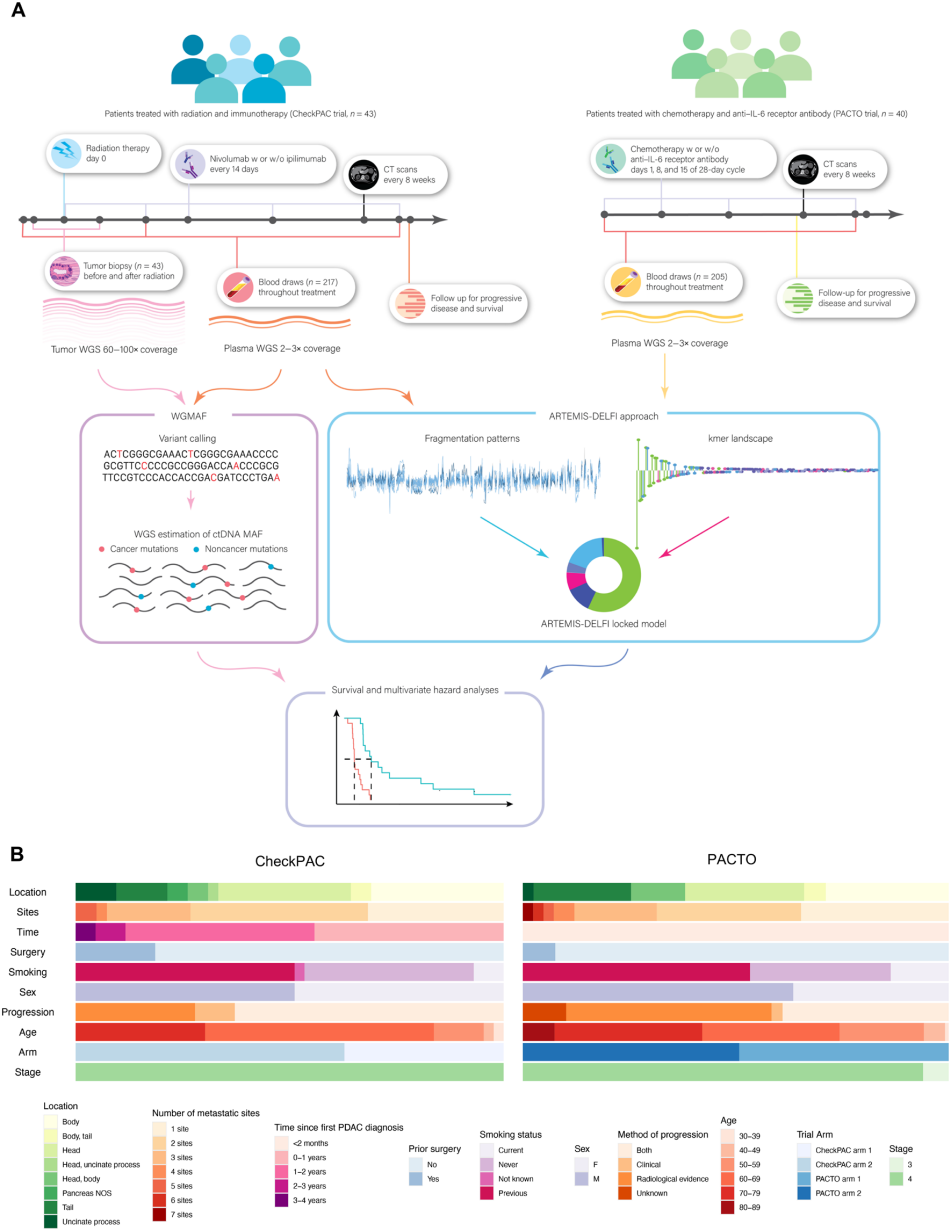
Overview of study design and samples. (**A**) Schematic of study design showing timeline of treatment, timeline of sample collection, and molecular analyses for both the CheckPAC and the PACTO cohorts. Tumor and WBCs were sequenced from patients in the CheckPAC cohort to generate a list of somatic variants present in tumor. WGMAF values were calculated in plasma samples as the number of mutant observations divided by total number of observations across all mutated positions with tumor-specific somatic mutations. ARTEMIS-DELFI scores were generated with a fixed model using fragmentation patterns, chromosomal gains and losses, and repeat elements as features. These scores were compared to progression-free survival and OS data, and multivariate hazard analyses were performed. (**B**) Description of CheckPAC and PACTO patients at baseline. Categories show the location of tumor, the number of metastatic sites, the time between initial diagnosis and trial enrollment, prior surgery, smoking status, sex, method of assessment of progression, trial arm, and cancer stage.

To evaluate methods for noninvasive prediction of survival and response to therapy, we analyzed a cohort of patients with pancreatic cancer, treated with radiation and immune checkpoint blockade, who had longitudinal blood collection in the CheckPAC trial (NCT02866383) (Fig. 1A) (*7*). Patients were eligible for the trial if they had unresectable refractory metastatic pancreatic cancer and had completed at least one line of standard-of-care chemotherapy. All patients received stereotactic body radiation therapy of 15 gray to one of their tumor sites and were randomized 1:1 to two arms: either nivolumab alone or nivolumab with ipilimumab. The patients’ tumors were biopsied before and after radiation, and plasma samples were obtained at regular intervals throughout therapy. A total of 84 patients were enrolled in this trial, of which 43 had tumor tissue or blood samples that were available for molecular analyses (Table 1 and fig. S1A). Tumors from patients included in our analyses were located in the body, head, tail, and uncinate process of the pancreas with up to five metastatic lesions detected at enrollment. The analyzed patients were diagnosed 4 months to more than 3 years before enrollment and a subset of patients (8 of 43) previously had surgical intervention for their disease. Responses were determined by a combination of clinical assessment and CT imaging using RECIST version 1.1, which compares images from any time point during therapy to baseline images. Patients included in our analyses had nearly equal representation of men and women (22 and 21, respectively), ranging in age from 35 to 75 years, and with most having a positive smoking history (Fig. 1B, Table 1, and table S1).

**Table 1.** Patient characteristics for CheckPAC and PACTO cohorts.

Patient characteristic	CheckPAC cohort		PACTO cohort	
	Analyzed	All	Analyzed	All
	***n* = 43**	***n* = 84**	***n* = 40**	***n* = 147**
**Age, years**				
Mean	65	63	65	65
Range	(35–75)	(35–80)	(36–84)	(36–84)
**Sex, *n* (%)**				
M	22 (51%)	44 (52%)	25 (63%)	86(59%)
F	21 (49%)	40 (48%)	15 (38%)	61 (41%)
**Time since first diagnosis (days)**				
Mean	476	93	18	25
Range	(93–1433)	(79–2072)	(10–47)	(7–525)
**Cancer stage, *n* (%)**				
II	-	-	-	1 (1%)
III	-	-	3 (8%)	14 (10%)
IV	43 (100%)	84 (100%)	37 (93%)	129 (88%)
Unknown	-	-	-	3 (2%)
**Histology, *n* (%)**				
Pancreatic ductal adenocarcinoma	41 (95%)	82 (98%)	36 (90%)	134 (91%)
Mucinous adenocarcinoma	-	-	3 (8%)	5 (3%)
Adenosquamous carcinoma	-	-	1 (3%)	5 (3%)
Unknown	2 (5%)	2 (2%)	-	3 (2%)
**Metastases, number**				
Mean	2	2	2	2
Range	(1–7)	(1–7)	(0–5)	(0–5)
**Location of metastases,*n* (%)**				
Liver	38 (88%)	80 (95%)	36 (90%)	107 (73%)
Lung	17 (40%)	26 (31%)	8 (20%)	33 (22%)
Mediastinal nodes	-	5 (6%)	1 (3%)	16 (11%)
Abdominal nodes	14 (33%)	23 (27%)	16 (40%)	51 (35%)
Peritoneal	4 (9%)	7 (8%)	12 (30%)	36 (24%)
Bone	2 (5%)	8 (10%)	2 (5%)	11 (7%)
Retroperitoneal	4 (9%)	15 (18%)	1 (3%)	13 (9%)
Ascites	7 (16%)	16 (19%)	2 (5%)	12 (8%)
Adrenal	1 (2%)	2 (3%)	2 (5%)	5 (3%)
**Trial arm, *n* (%)**				
Nivo	16 (37%)	41 (49%)	-	-
Nivo + ipi	27 (63%)	43 (51%)	-	-
Chemo	-	-	20 (50%)	70 (48%)
Chemo + IL-6	-	-	20 (50%)	71 (48%)
N/A	-	-	-	6 (4%)
**Site of radiotherapy, *n* (%)**				-
Liver	34 (79%)	64 (76%)	-	-
Lung	3 (7%)	5 (6%)	-	-
Abdominal wall	1 (2%)	1 (1%)	-	-
Local recurrence	3 (7%)	9 (11%)	-	-
Retroperitoneal	-	1 (1%)	-	-
Other	2 (5%)	4 (5%)	-	-
**Overall survival, days**				
Mean	322	215	280	301
Range	(37–2720)	(10–2720)	(25–1059)	(9–1187)
**Progression-free survival, days**				
Mean	139	91	168	180
Range	(26–1433)	(4–1433)	(12–678)	(9–678)
**Best overall response, BOR, *n* (%)**				
Partial response (PR)	7 (16%)	7 (8%)	14 (35%)	53 (36%)
Stable disease (SD)	13 (30%)	16 (19%)	16 (40%)	50 (34%)
Progressive disease (PD)	21 (49%)	51 (61%)	6 (15%)	26 (18%)
Not evaluable (NE)	2 (5%)	10 (12%)	4 (10%)	18 (12%)
**Survival status, *n* (%)**				
Dead	42 (98%)	83 (99%)	40 (100%)	147 (100%)
Alive	1 (2%)	1 (1%)	-	-
**Mutations, *n* (%)**				
*KRAS*	26 (60%)	-	36 (90%)	-
*TP53*	24 (56%)	-	32 (80%)	-
*SMAD4*	4 (9%)	-	-	-
*CDKN2A*	5 (12%)	-	6 (15%)	-

To assess these noninvasive approaches for monitoring in an independent validation study, we analyzed individuals from the PACTO trial (NCT02767557) (*33*). Patients were enrolled in the PACTO trial if they had locally advanced or metastatic pancreatic adenocarcinoma and had received no prior antineoplastic chemotherapy or anticancer drugs. Patients were randomized 1:1 to two arms, both of which received gemcitabine/nab-paclitaxel, while one arm also received the IL-6 receptor antibody tocilizumab. Patients from both arms had plasma samples taken at regular intervals throughout therapy. The trial enrolled 147 treatment-naïve patients with pancreatic cancer, of which a subset of 40 with known ctDNA levels at baseline were included in our analyses (Fig. 1A, Table 1, and fig. S1A). The characteristics of the analyzed patients in the PACTO trial were largely similar to those of the CheckPAC trial but had a slightly older age profile, no previous systemic treatment after first PDAC diagnosis, and included three patients with locally advanced but not metastatic disease. (Fig. 1B, Table 1, and table S2).

We assessed whether response to therapy in patients from these trials could be evaluated using both tumor-informed as well as tumor-independent methods for predicting response and survival. We compared these approaches to current methods of measuring response to therapy using standard imaging approaches (RECIST 1.1), as well as the known serum pancreatic cancer biomarker CA19-9. For all the molecular methods, we evaluated blood draws for patients in CheckPAC from the baseline time point (*n* = 35) as well as the second time point at a median of 8 weeks after initiation of therapy (*n* = 33, median = 54 days, range = 14 to 114 days) with similar time points evaluated in PACTO. This allowed for a direct comparison to 8-week RECIST 1.1 measurements (median: 49 days, range = 26 to 60 days) (fig. S2A and table S1). Previous studies have suggested that this time point would be useful to assess for response using liquid biopsy approaches for patients treated with immune checkpoint blockade (*34*, *35*).

### Tumor-informed WGMAF approach

We developed a tumor-informed approach (WGMAF) based on WGS of the tumors from these patients (table S3). In this approach, somatic mutations were detected in the tumor using the matched buffy coat (normal) as a filter to select high-quality somatic variants. The tumor-derived somatic mutations from each patient were then evaluated as a set in the cfDNA from that patient’s plasma. For every plasma sample, we counted the total number of fragments containing mutations in the set and divided this by the total number of fragments at these locations. This estimate of MAF does not rely on a panel of mutations or known variants and can therefore be optimized for each patient (fig. S3).

**Fig. 2.**
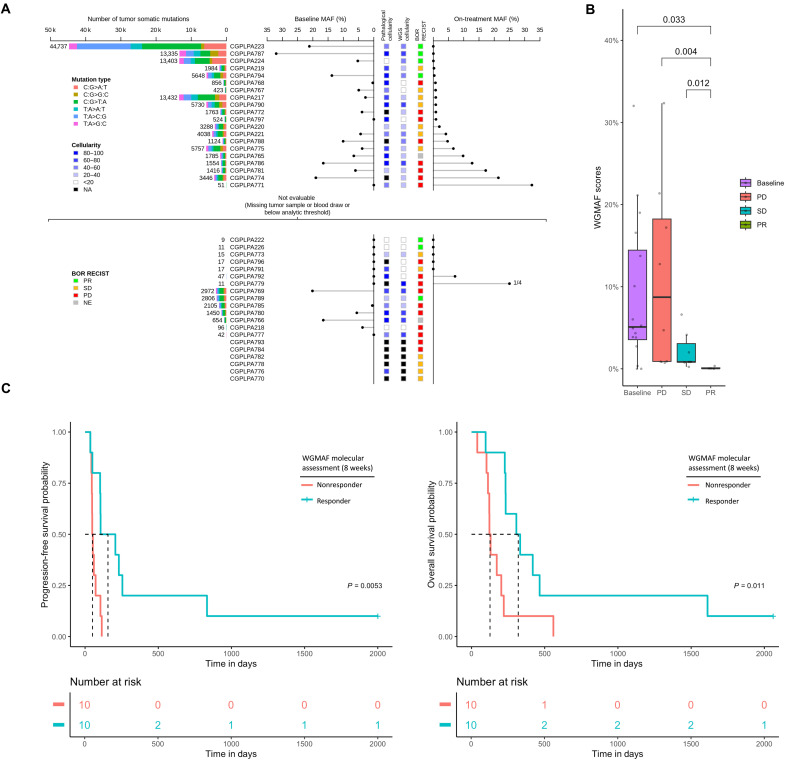
WGMAF approach predicts survival for patients in CheckPAC trial. (**A**) Tree plot showing WGMAF levels at baseline and on-treatment as well as tumor cellularity measurements and clinical response for each patient. The section above the dashed line describes evaluable patients, while the section below the dashed line shows patients who were not evaluable because of unavailable tumor or plasma samples or an insufficient number of somatic mutations found in tumor. The number of somatic mutations identified in tumors are listed to the left of the tree plot in descending order, colored by mutation type. Baseline MAF is plotted on the left side of tree plot, and on-treatment MAF is plotted on the right side. The central spine of the tree plot shows the tumor cellularity using two methods, pathological cellularity evaluated from hematoxylin and eosin sample and cellularity assessed from molecular sequencing data, as well as BOR RECIST for each patient. (**B**) Boxplot of WGMAF values for baseline samples and follow-up samples sorted by clinical PD, SD, and PR. *P* values were calculated using the Wilcoxon signed-rank test. (**C**) Kaplan-Meier survival curves showing progression-free survival probability and OS probability based on the median WGMAF value for CheckPAC patients at the second follow-up (on-treatment) plasma evaluation. Nonresponders were classified as those with WGMAF above median value of 0.87%, while responders were classified as those with WGMAF below 0.87%.

To assess our WGMAF approach for cancer monitoring, we performed WGS using tumor and matched normal samples (target coverage 60 to 100× for tumors and 30 to 60× for normal samples) for all patients where samples were available and where tumors had pathologically assessed neoplastic cellularity >20% (*n* = 37) (fig. S1). Comparison of sequence alterations in the matched tumor and normal samples revealed 9 to 13,432 alterations among these samples as well as one tumor with hypermutator phenotype harboring 44,737 somatic mutations (Fig. 2A, fig. S4, and table S4). Analysis of the somatic alterations revealed that 82% of the tumors sequenced harbored a mutation in the *KRAS* oncogene and 75% harbored a mutation in the *TP53* tumor suppressor gene (Table 1, fig. S4, and table S4), both of which are commonly mutated in PDAC (*36*). The tumor with a hypermutator phenotype harbored a G440E mutation in *MLH1*, which was classified as a likely pathogenic germline change by InSiGHT and ClinVAR (*37*, *38*), but has only been documented as a somatic change in two cancer samples in the COSMIC database (*39*). None of these patients were found to have mismatch repair deficiency by immunohistochemistry.

The evaluability of patient samples by WGMAF requires a sufficient number of somatic mutations identified in the sequenced tumor to enable detection of ctDNA in plasma even when the tumor fraction in plasma is low. Here, we considered a patient evaluable by this approach if at least 50 mutations were identified in the tumor as the probability of identifying one or more somatic mutation in plasma is 0.75 or higher when the tumor fraction is 1% or higher (table S5). Of the 25 patients who were evaluable by WGMAF, 20 patients had a second blood draw at 8 weeks from treatment initiation, allowing these patients to be examined for both cfDNA and imaging analyses (fig. S2, A and B). To independently determine the accuracy of the WGMAF approach, we compared the WGMAF estimated level of ctDNA for 10 samples using this approach to a targeted sequencing approach of 64 genes for determining the MAF and found that the two methods had a high correlation across a range of MAF values (*R* = 0.90, Pearson correlation, *P* < 0.0001, Wilcoxon signed-rank test) (fig. S5).

As WGMAF was highly correlated with targeted sequencing approaches that directly measure MAF, we next evaluated whether a landmark analysis of WGMAF after therapy was associated with clinical response. We chose to focus on landmark analyses as they have the advantage of being logistically simpler (only a single blood tube is needed), and there is precedent for using landmark measurements for noninvasive determination of therapeutic response (*40*, *41*). While baseline samples of these patients (*n* = 18) typically had relatively high WGMAF scores, with a median value of 4.9% [95% confidence interval (CI) = 1.9 to 10%] (Fig. 2B), landmark samples at the 8-week time point revealed very low on-treatment WGMAF measurements (median score = 0.044%, 95% CI = 0.0048 to 0.53%) for patients who were determined to be partial responders (*n* = 4) from their best overall responses (BORs) by RECIST 1.1, whereas patients with stable disease (*n* = 7) (median score = 0.84%, 95% CI = 0.046 to 3.8%) or progressive disease (*n* = 8) (median score = 8.7%, 95% CI = 1.34 to 19.4%) had higher WGMAF scores [Wilcoxon signed-rank test *P* = 0.004 for PR (partial response) compared to PD (progressive disease), *P* = 0.012 for PR compared to SD (stable disease)] (Fig. 2, A and B).

Using the median on-treatment landmark WGMAF value as a cut point for determining molecular response, we found that of the 20 patients analyzed (fig. S1A), the 10 patients with scores below the cut point identified as molecular responders had a statistically longer progression free survival (PFS) compared to patients with scores above the cut point identified as molecular nonresponders (PFS of 157 days versus 51 days, respectively) [hazard ratio (HR) = 0.23, 95% CI = 0.07 to 0.69, *P* = 0.0053, log-rank test]. Similarly, molecular responders had a significantly longer overall survival (OS) compared to molecular nonresponders (OS of 319 days compared to 126 days, respectively (HR = 0.29, 95% CI = 0.11 to 0.79, *P* = 0.011, log-rank test) (Fig. 2C). Among patients with stable disease by imaging at the 8-week time point (*n* = 9) molecular response assessment by WGMAF provided a similar stratification of OS, with molecular responders having a median OS of 332 days compared to 112 days for molecular nonresponders (HR = 0.16, 95% CI = 0.02 to 1.16, *P* = 0.0019, log-rank test) (fig. S6).

To assess whether WGMAF scores are an independent predictor of OS when evaluated together with known clinical risk factors of pancreatic cancer progression, we performed a multivariate analysis with factors known to be associated with outcomes for patients with pancreatic cancer (*42*, *43*). We included age, Eastern Cooperative Oncology Group performance status (ECOG PS), weight loss, modified Glasgow prognostic score (MGPS), and treatment type, as well as the standardized WGMAF scores before and after treatment initiation. In this multivariate analysis, we found WGMAF scores were the only statistically significant feature associated with patient OS (HR = 2.38, 95% CI = 1.20 to 4.73, *P* = 0.01, Cox proportional hazards) (fig. S7).

### Genome-wide cfDNA fragmentation analyses

We next evaluated the recently developed genome-wide fragmentome approach (*26*) as a molecular tool for detecting response to therapy in these same patients with metastatic pancreatic cancer. We have previously shown that genome-wide analyses of cfDNA can be used to evaluate underlying genomic, chromatin, and epigenomic changes specific to cancer (*26*, *27*, *44*). To evaluate cfDNA fragmentation in the plasma from patients in the CheckPAC trial, we examined fragmentation profiles reflecting the size and distribution of tens of millions of fragments in 473 nonoverlapping 5-Mb regions and spanning approximately 2.4 Gb of the genome. Analysis of the fraction of short [100 to 150 base pairs (bp)] to long (151 to 220 bp) cfDNA fragments in each of 473 5-Mb bins revealed greater heterogeneity within patients at baseline or with disease progression as compared to those who experienced stable disease or a partial response (fig. S8).

**Fig. 3.**
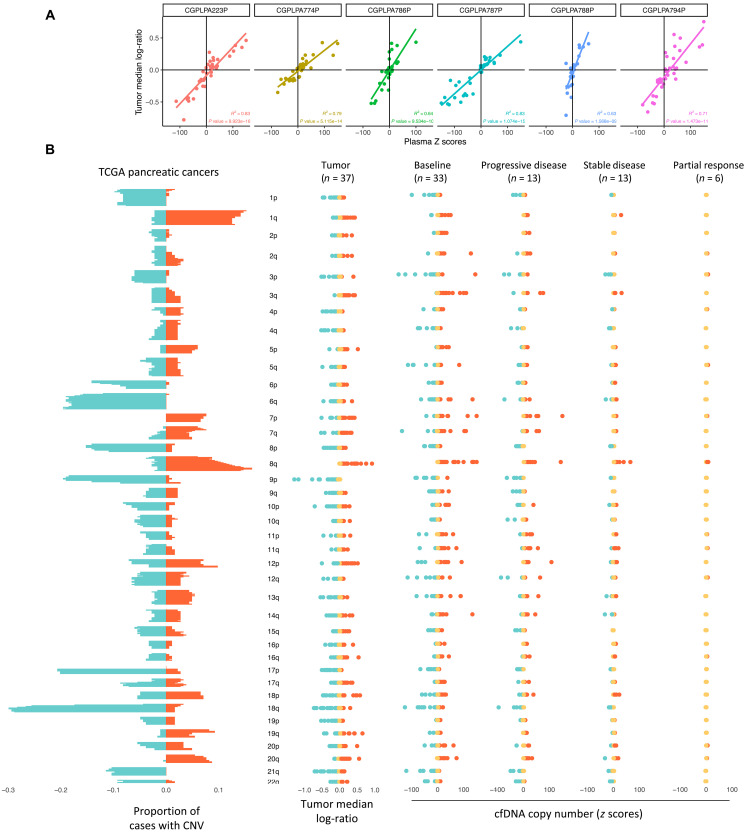
cfDNA fragmentation features reflect underlying tumor biology in pancreatic cancer. (**A**) Changes in the cell-free chromosomal arm content in the circulation (plasma *z* scores) compared to the changes in chromosome arm content in tumors (tumor median log ratio) for patients who had greater than 10% plasma MAF by WGMAF. (**B**) Fraction of cases with copy number variation (CNV) changes by chromosomal arm for PDAC cancers in TCGA are shown alongside assessment of copy number changes in CheckPAC patients, as quantified by the median log ratio by arm for tumor copy number gains and losses, as well as *z* scores by arm for all patients with plasma samples at baseline and at follow-up for each of the clinical RECIST 1.1 response categories.

As the cfDNA fragmentome may comprise changes related to large-scale genomic changes released from cancer cells (*26*), we also examined chromosomal gains and losses in the circulation of these patients. For samples obtained at the baseline time point with ctDNA levels above 10% as quantified by WGMAF, we found statistically significant correlations of arm chromosomal gains and losses in plasma and matched tumor (all correlations with correlation coefficient > 0.64 and *P* < 0.0001) (Fig. 3A). The paired plasma-tumor copy number changes reflected those commonly found in pancreatic cancer including gains in 1q, 8q, 5p, and 7p as well as losses in 1p, 3p, 6p, 6q, 9p, 17p, and 18q (Fig. 3B) (*45*, *46*). We found that, at the on-treatment time point, patients with partial clinical response had fewer copy number alterations than those with stable disease, who in turn had fewer than those with progressive disease (Fig. 3B). These analyses demonstrate not only that chromosomal changes found in the circulation reflect the known biology of pancreatic cancer but that they may also reflect changes in tumor burden in response to therapy (Fig. 3B).

**Fig. 4.**
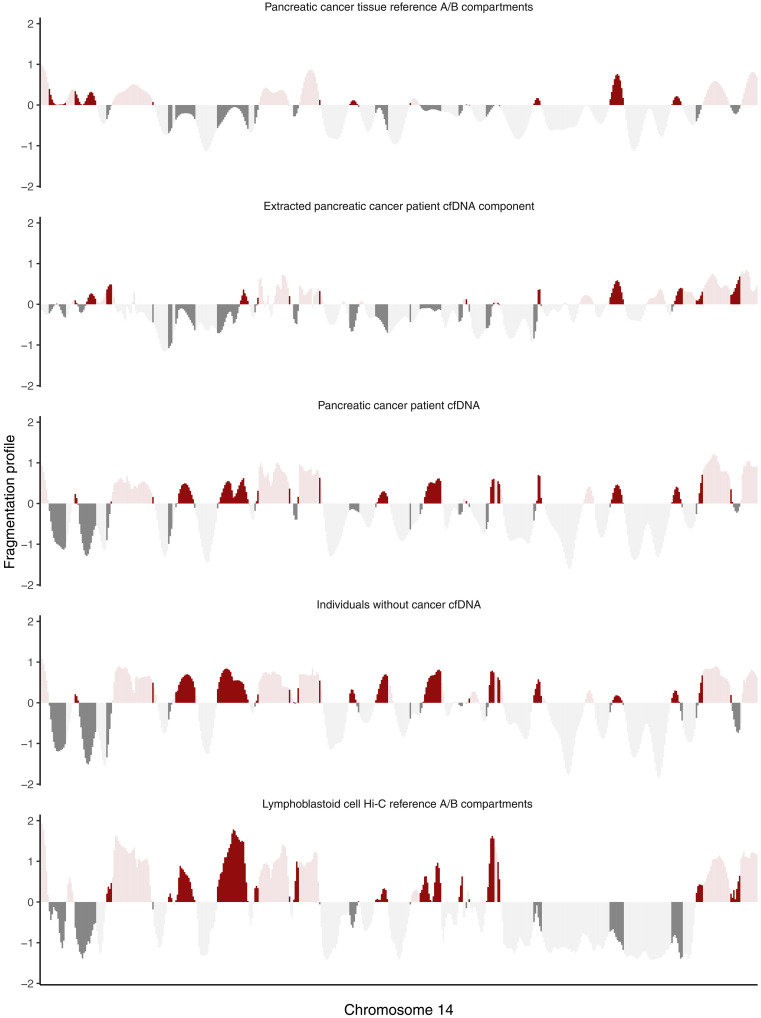
Genome-wide cfDNA fragmentation profiles comprise chromatin structures from peripheral blood cells and pancreatic cancer. Comparison of plasma fragmentation features to reference A/B compartments across chromosome 14. The first panel shows chromatin A/B compartments derived from pancreatic cancer tissue methylation (*36*). The second panel shows a median deconvoluted pancreatic cancer component based on the six samples with the highest ctDNA levels. The third panel shows the median fragmentation profile in the plasma for those samples, and the fourth panel shows the median fragmentation profile for a set of healthy plasma controls. The final panel shows chromatin A/B compartments for lymphoblast cells. Dark shading indicates regions of the genome where the two reference tracks are discordant in domain (open/closed) or magnitude. The extracted pancreatic cancer component has the greatest similarity to the pancreatic cancer reference track, and the healthy plasma has the greatest similarity to the lymphoblast reference track.

To examine the contribution of pancreatic cancer to the cfDNA, we compared high-throughput sequencing chromosome conformation capture open (A) and closed (B) compartments of reference pancreatic cancers and lymphoblastoid samples (*36*) to plasma samples from healthy individuals or those within the CheckPAC trial (Fig. 4) (*47*). Assessing the six patient samples with the highest ctDNA tumor burden revealed that these contained contributions that were similar to healthy individuals as well as a one that related to the cancer samples. While healthy control plasma samples were highly correlated to lymphoblastoid cell lines, pancreatic cancer plasma samples and the extracted pancreatic cancer component were more similar to the pancreatic cancer A/B compartments. These analyses demonstrate that cfDNA from patient samples in the CheckPAC trial reflects a combination of chromatin compartments from both peripheral blood cells and pancreatic cancer.

**Fig. 5.**
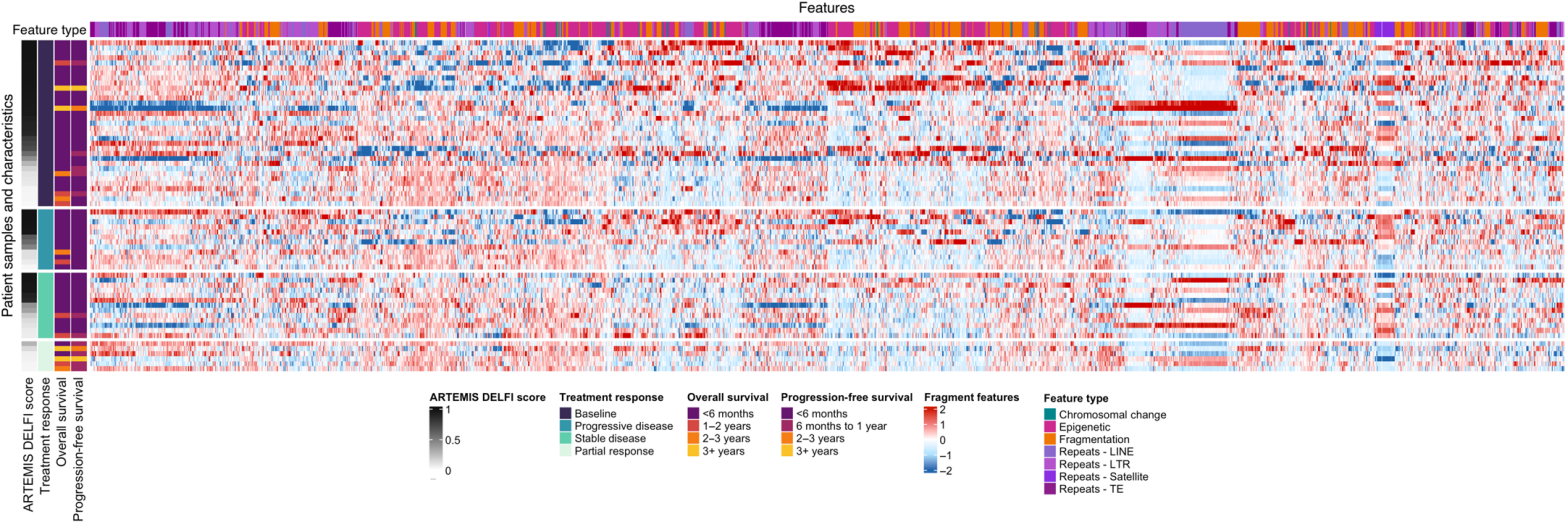
Heatmap of clinical features and cfDNA fragmentation and genomic repeat features. The vertical axis is categorized by all patients with plasma samples at baseline and at follow-up for each of the clinical RECIST 1.1 response categories and sorted by ARTEMIS-DELFI scores in descending order. All molecular features evaluated are plotted along the horizontal axis and colored by feature type. The heatmap color scale reflects the deviation of cfDNA features as compared to the mean of noncancer individuals.

We have previously shown that fragmentation patterns analyzed by DELFI are also influenced by structural and epigenetic changes in genome-wide repeat elements including long interspersed nuclear elements, short interspersed nuclear elements, long terminal repeats, and satellites and transposable elements and could be assessed using ARTEMIS, an alignment-free method allowing granular assessment of repeat elements in low-coverage WGS of cfDNA (*32*). The multitude of fragmentation, chromosomal, epigenetic, and repeat landscape features showed widespread changes among the CheckPAC samples analyzed (Fig. 5). A locked ensemble model of 2343 features from the ARTEMIS and DELFI fragmentomic approaches (*32*) was used to generate a joint ARTEMIS-DELFI score for all CheckPAC samples (fig. S9 and table S5), reflecting the probability that an individual’s plasma sample contains tumor derived DNA.

**Fig. 6.**
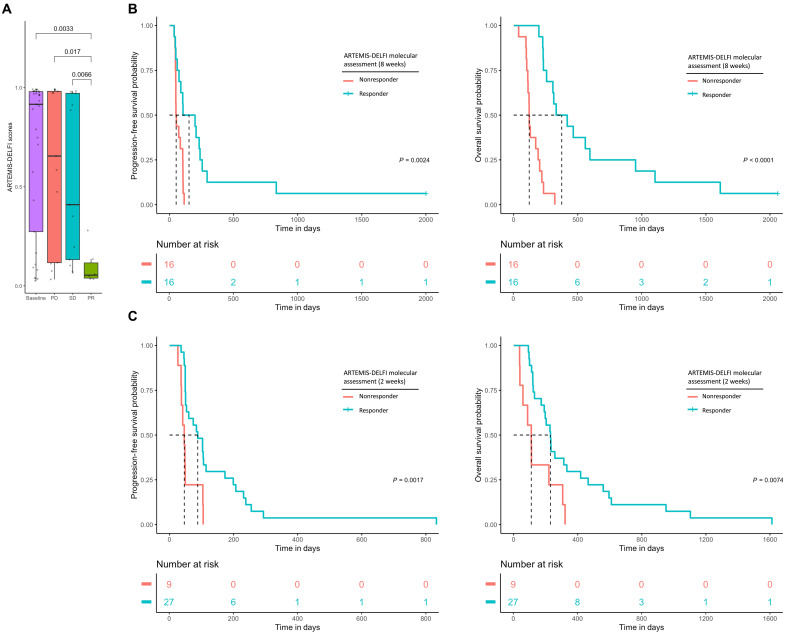
ARTEMIS-DELFI scores predict survival for patients in CheckPAC trial. (**A**) Boxplot of ARTEMIS-DELFI scores for all patients with plasma samples at baseline, and at follow-up for each of the clinical RECIST 1.1 response categories. (**B**) Kaplan-Meier curves of progression-free survival probability and OS probability based on median landmark ARTEMIS-DELFI score at 8 weeks. Patients are classified as responders or nonresponders if follow-up ARTEMIS-DELFI scores are below or above the median follow-up score, respectively. (**C**) Kaplan-Meier curves of progression-free survival probability and OS probability based on “fast-fail” landmark ARTEMIS-DELFI score after one cycle of treatment (2 weeks). Patients are classified as responders or nonresponders if follow-up ARTEMIS-DELFI scores are below or above the median follow-up score, respectively

While baseline samples of CheckPAC patients (*n* = 36) had relatively high ARTEMIS-DELFI scores, with a median score of 0.93 (95% CI = 0.65 to 0.93) (Fig. 6A), landmark samples at the 8-week time point revealed very low on-treatment ARTEMIS-DELFI measurements (median score = 0.15, 95% CI = 0.075 to 0.38) for patients who were determined to be partial responders (*n* = 6) using BORs by RECIST 1.1, whereas patients with stable disease (*n* = 11) (median SD score = 0.38, 95% CI = 0.30 to 0.81) or progressive disease (*n* = 15) (median PD scores = 0.68, 95% CI = 0.45 to 0.90) had higher ARTEMIS-DELFI scores (*P* < 0.017 for all comparisons, Wilcoxon signed-rank test) (Fig. 6A and figs. S1 and S10).

Using the median on-treatment landmark ARTEMIS-DELFI score as a cut point for determining molecular response, we found that of the 32 patients analyzed (fig. S1A), 16 patients with scores below the cut point identified as molecular responders had a significantly longer PFS compared to 16 patients with scores above the cut point identified as molecular nonresponders (PFS of 105 days versus 53 days, respectively) (HR = 0.28, 95% CI = 0.12 to 0.67 *P* = 0.0024, log-rank test). Similarly, molecular responders had a significantly longer OS compared to molecular nonresponders (OS of 233 days versus 172 days, respectively) (HR = 0.12, 95% CI = 0.046 to 0.31, *P* < 0.0001, log-rank test) (Fig. 6B). For patients with stable disease by imaging at the 8-week time point (*n* = 16), molecular response assessment by ARTEMIS-DELFI provided a similar stratification of OS, with molecular responders having a median OS of 399 days compared to 157 days for molecular nonresponders (HR = 0.26, 95% CI = 0.071 to 0.93, *P* = 0.0025, log-rank test) (fig. S11). These findings suggested that ARTEMIS-DELFI scores were significant predictors of outcome for patients with pancreatic cancer after initiation of immune checkpoint blockade.

**Fig. 7.**
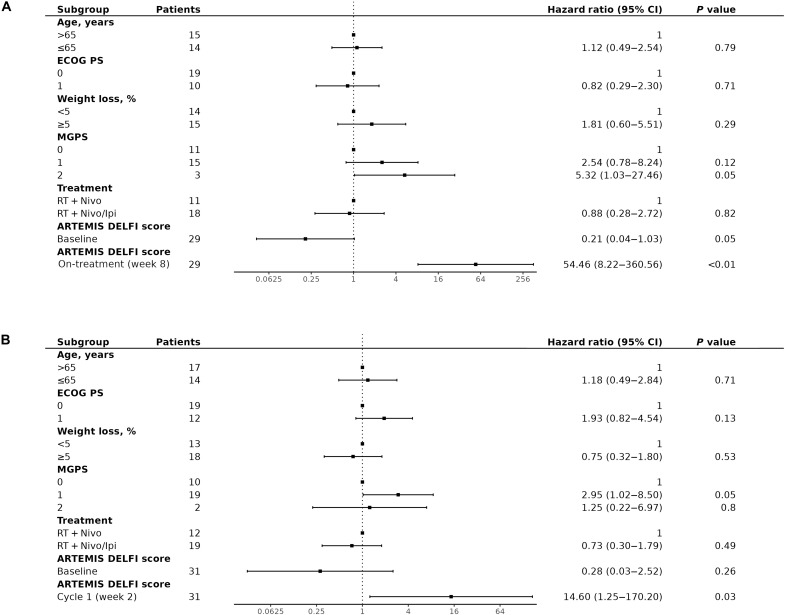
Multivariate hazard analyses demonstrate on-treatment ARTEMIS-DELFI scores as independent predictors of OS for patients in the CheckPAC trial. Multivariate Cox proportional hazard analyses were generated for each molecular method and fit to OS adjusting for clinical subgroups. Each of the indicated subgroups that have been shown to be significant on univariate analyses in previous studies (*7*) have been included in the multivariate analysis. Hazard models are shown using (**A**) ARTEMIS-DELFI scores at baseline and 8-week follow-up values for patients in the CheckPAC study and (**B**) ARTEMIS-DELFI scores at baseline and after one cycle of treatment for patients in the CheckPAC study.

To assess whether ARTEMIS-DELFI scores were independent predictors of OS when examined together with clinical risk factors of pancreatic cancer progression, we performed a multivariate analysis with factors known to be associated with outcomes for patients with pancreatic cancer (*42*, *43*) similar to the WGMAF analysis described earlier. In this multivariate analysis, we found that on-treatment, ARTEMIS-DELFI scores were significantly associated with patient OS (HR = 55, 95% CI = 8.2 to 361, *P* < 0.0001, Cox proportional hazards) (Fig. 7A), while clinical characteristics did not affect outcome.

We evaluated whether an analysis of patients with high ARTEMIS-DELFI scores (above the 75th percentile) at a 2-week time point after initiation of therapy (*n* = 9) would allow for a rapid determination of response (“fast fail”). We found that patients with scores above this threshold had a significantly shorter median PFS and OS than patients with lower scores (*n* = 27) (PFS of 48 days versus 88 days, respectively; HR = 0.29, 95% CI = 0.17 to 0.66, *P* = 0.0017, log-rank test) (OS of 110 days versus 230 days, respectively; HR = 0.35, 95% CI = 0.16 to 0.78, *P* = 0.0074, log-rank test) (Fig. 6B), and remained significant even in multivariate analysis (HR = 14.60, 95% CI = 1.25 to 170.20, *P* = 0.03, Cox proportional hazards) (Fig. 7B). These findings suggested that ARTEMIS-DELFI scores were potentially useful predictors of outcome for patients with pancreatic cancer even after one treatment cycle of immune checkpoint blockade.

### Comparison to other methodologies of molecular response assessment

We examined how the molecular approaches described above compared to each other as well as existing methodologies for evaluating tumor burden, including RECIST 1.1 classification, CA19-9 serum measurement, and ichorCNA (*20*). We first compared ARTEMIS-DELFI and WGMAF scores to each other from across all evaluable time points and found that they were highly correlated (*R* = 0.84, Pearson correlation, *P* < 2.2 × 10^−16^, Wilcoxon signed-rank test) (fig. S12). While all patients in the CheckPAC trial had cancer, we considered plasma samples from patient CGPLPA223 as not harboring any ctDNA after this patient had a complete long-term response and use the genome-wide MAF scores in these samples to determine the limit of blank ARTEMIS-DELFI. The median WGMAF score for these ctDNA negative samples was 0.0081% (range less than 0.0069 to 0.028%) corresponding to an ARTEMIS-DELFI score of 0.045 (table S5). Using the range of ctDNA MAF values obtained by WGMAF in the remaining patient samples, we determined that 83% of samples tested positive with ARTEMIS-DELFI when WGMAF values were ≥0.001 (fig. S13).

In contrast to these approaches, RECIST 1.1 classification of the first follow-up CT scan did not stratify patients in the CheckPAC trial with respect to their OS, with patients classified as PR, PD, or SD having similar outcomes (*P* = 0.15, log-rank test). In contrast, RECIST measurements for BOR ultimately stratified patients for OS (*P* = 0.014, log-rank test) (fig. S14). For CA19-9, we found that after removing four patients with CA19-9 <37 U/ml at baseline (patients whose tumors do not secrete CA19-9) (*48*), the remaining patients who were clinical responders had a statistically significant lower levels of this protein at an equivalent 8-week time point (fig. S15) (median CA19-9 of patients with partial response = 177 U/ml, 95% CI: 12 to 1300 U/ml) compared to those with progressive disease at follow-up (median CA19-9 of patients with progressive disease = 5630 U/ml, 95% CI = 1400 to 26,000 U/ml, *P* = 0.0046, Wilcoxon signed-rank test) (fig. S16A). However, patients with clinical response did not have a significant difference in mean CA19-9 levels compared to patients with stable disease or patients at baseline (median CA19-9 of patients with stable disease = 882 U/ml, 95% CI: 250 to 6800 U/ml, *P* = 0.18, Wilcoxon signed-rank test) (fig. S16B). Similarly for patients with tumor levels quantified by ichorCNA, we found that at the 8-week time point, there was no significant difference between individuals with a partial response (median ichorCNA MAF = 1.4%, 95% CI: 0.12 to 1.6%) and those with progressive disease (median ichorCNA MAF = 2.2%, 95% CI: 1.9 to 12%, *P* = 0.34, Wilcoxon signed-rank test). However, patients with partial response had significantly lower tumor fractions than those with stable disease [ichorCNA tumor fraction = 4.9% (95% CI = 2.5 to 5.3%)] (*P* = 0.041, Wilcoxon signed-rank test) (fig. S17).

We also compared ARTEMIS-DELFI or WGMAF scores with CA19-9 measurements for individuals who were CA19-9 secretors and found that although there was significant correlation with each of these with CA19-9 [for ARTEMIS-DELFI *R* = 0.49 (Pearson correlation), *P* < 0.0001, Wilcoxon signed-rank test and for WGMAF *R* = 0.55 (Pearson correlation), *P* < 0.0001, Wilcoxon signed-rank test], it was weaker than when WGMAF and ARTEMIS-DELFI were compared to each other, and that for patients who were nonsecretors, there was no correlation (for ARTEMIS-DELFI *R* = −0.021, *P* = 0.95, Wilcoxon signed-rank test and for WGMAF *R* = 0.27, *P* = 0.66, Wilcoxon signed-rank test) (fig. S18). Overall, these analyses suggest that existing approaches may be inadequate for accurate determination of response to therapy in patients with pancreatic cancer.

**Fig. 8.**
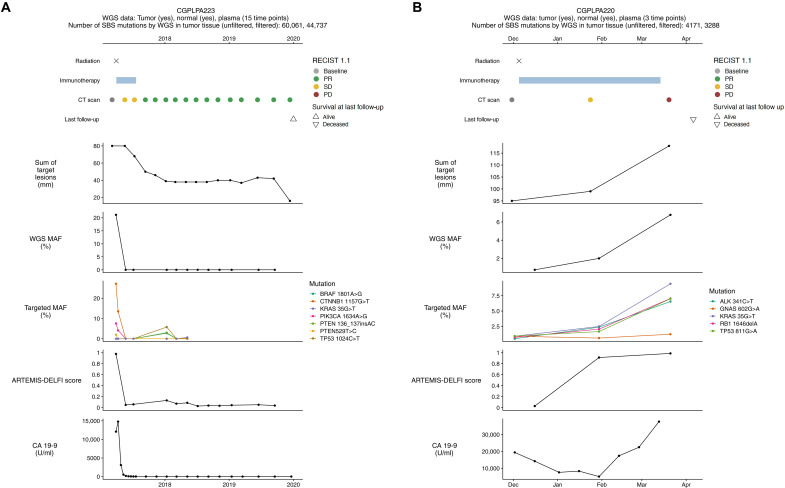
Example of a molecular responder and nonresponder to treatment using different methodologies. Response to treatment is shown for a patient with clinical BOR of partial response (**A**) and stable disease (**B**). Patient clinical paths are shown in the top panels, followed by methodologies for monitoring response to treatment. For the top panels, patient treatment is indicated in the top two rows, radiologic assessments are plotted in the third row, and the last follow-up is indicated in the fourth row. Sum of target lesions were assessed from standard-of-care CT scans. WGMAF, targeted MAF, and ARTEMIS-DELFI scores are plotted for each of the blood draw time points. CA19-9 was measured clinically at regular intervals throughout treatment.

We compared longitudinal profiles for all patients enrolled in the CheckPAC trial, showing the dynamics of WGMAF and ARTEMIS-DELFI scores over time as compared to RECIST scoring, lesion size, targeted MAF, and CA19-9 measurements (fig. S19). WGMAF could not be performed in 14 patients either due to lack of suitable tumor biopsy or insufficient mutations observed after whole-genome analyses, while ARTEMIS-DELFI analyses were performed in all 40 patients. Analyzable patients could largely be categorized as either molecular responders or nonresponders. As examples, patient CGPLPA223 had a molecular response at 8 weeks, while patient CGPLPA220 was a molecular nonresponder at the same time point (Fig. 8). For both patients, the first follow-up CT scan at this 8-week time point indicated stable disease. For patient CGPLPA223, the partial response was ultimately observed at the third follow-up CT scan (week 24), while for patient CGPLPA220, progressive disease was indicated at the next scan (week 15).

Although landmark studies at the 8-week time point have logistical advantages of only requiring a single sample for analysis, we also used the WGMAF and a variation of the DELFI approach to compare changes in ctDNA over time. Evaluation of any increases or decreases in WGMAF levels from baseline to the 8-week time point resulted in a significant difference in progression-free survival (*P* = 0.018, log-rank test) but not OS (*P* = 0.16, log-rank test) for patients who were evaluated using this approach (*n* = 17) (fig. S20). Although ARTEMIS-DELFI scores are not linearly related to MAF levels and therefore cannot be used directly for quantitative measurement of MAF levels over time, we used a variation of DELFI (*31*), called DELFI-TF, which was specifically designed to measure changes in ctDNA levels, and found that grouping patients by increasing (*n* = 8) or decreasing (*n* = 20) DELFI-TF scores between baseline and the 8-week time point was predictive of OS (*P* = 0.0047, log-rank test) and PFS (*P* = 0.00012, log-rank test) in the patients (*n* = 28) where samples were available (fig. S21).

### Validation in external cohort

**Fig. 9.**
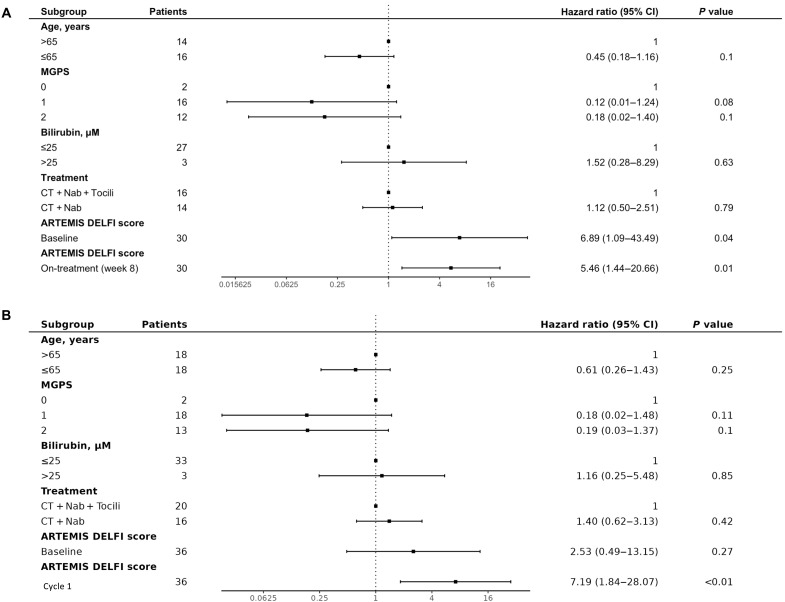
Multivariate hazard analyses demonstrate on-treatment ARTEMIS-DELFI scores as independent predictors of OS for patients in the PACTO trial. Multivariate Cox proportional hazard analyses were generated for each molecular method and fit to OS adjusting for clinical subgroups. Each of the indicated subgroups that have been shown to be significant on univariate analyses were included in the multivariate analysis. Hazard models are shown using (**A**) ARTEMIS-DELFI scores at baseline and 8-week follow-up for patients in the PACTO study and (**B**) ARTEMIS-DELFI scores at baseline and after one cycle of treatment for patients in the PACTO study.

We assessed whether our liquid biopsy approach would provide a molecular stratification predictive of clinical end points in the PACTO trial. As this trial did not collect tumor tissue, we could only examine the ARTEMIS-DELFI approach and evaluated the same locked ARTEMIS-DELFI model at the same 8-week time point evaluated in the CheckPAC cohort, however using median on-treatment score from PACTO as cut point (figs. S9, S21, and S22, and table S6). Molecular responders had a significantly longer OS than nonresponders (OS: 288 days compared to 199 days, HR = 0.488, 95% CI = 0.24 to 1.01, *P* = 0.048, log-rank test) (fig. S23A and table S6). We found a trend in PFS differences between patients with molecular response (*n* = 16) compared to those without molecular response (*n* = 16) (PFS: 207 days compared to 139 days, respectively) (HR = 0.54, 95% CI = 0.26 to 1.14, *P* = 0.096, log-rank test) (fig. S23 and table S6). A multivariate hazard analysis of this cohort revealed that the on-treatment ARTEMIS-DELFI score was a statistically significant predictor of OS (HR = 5.46, 95% CI = 1.44 to 20.66, *P* = 0.01, log-rank test). (Fig. 9A). Imaging at this time point revealed that RECIST 1.1 assessment was not associated with OS (fig. S24). These results suggest that the association between changes in ARTEMIS-DELFI scores and patient survival during therapy are generalizable across treatment settings for patients with late-stage pancreatic cancer.

We evaluated whether the fast fail analysis of patients with high ARTEMIS-DELFI scores (above the 75th percentile) after one cycle of treatment (4-week time point) in this cohort (*n* = 9) would allow for a rapid determination of response. We found that patients with scores above this threshold had a significantly shorter median PFS and OS than patients with lower scores (*n* = 27) (PFS of 106 days versus 196 days respectively, HR = 0.27, 95% CI = 0.11 to 0.67, *P* = 0.0027, log-rank test), (OS of 151 days versus 280 days respectively, HR = 0.17, 95% CI = 0.064 to 0.46, *P* = 0.00011, log-rank test) (fig. S23B), and remained significant even in a multivariate analysis (HR = 7.19, 95% CI = 1.84 to 28.07, *P* < 0.01, Cox proportional hazards) (Fig. 9B). These findings validated that ARTEMIS-DELFI scores were potentially useful early predictors of outcome for patients with pancreatic cancer.

## DISCUSSION

In this study, we developed and evaluated two approaches for noninvasive cfDNA monitoring for patients with pancreatic cancer: a tumor-informed mutation-based approach (WGMAF) and a tumor-independent fragmentation-based approach (ARTEMIS-DELFI). We found that both WGMAF and ARTEMIS-DELFI scores after initiation of therapy were significantly associated with PFS and OS in the CheckPAC trial. In addition, the ARTEMIS-DELFI approach was successfully validated in the plasma-only PACTO trial. These approaches were better predictors of outcome than imaging or other existing clinical and molecular markers at 2 months after treatment initiation.

The tumor- and mutation-informed WGMAF methodology provides a unique approach to mutation-based analyses of tumor-derived cfDNA in the circulation. By taking advantage of knowledge of the specific mutations in a patient’s tumor, the approach can be personalized for monitoring an individual patient’s cancer. Use of a genome-wide approach permits the laboratory processes to be standardized across patients and does not require development of new PCR or mutation-related probes for each patient as is necessary for other methods (*49*). This approach is not cancer-type specific and has the potential to be applied in a variety of treatment settings. A clear weakness of the approach, however, is that it requires a tumor biopsy and sufficient tumor-derived DNA and, therefore, may not be applicable for all patients. In the CheckPAC trial of patients with advanced pancreatic cancer, half of the patients could not be analyzed because of these limitations.

In contrast to WGMAF, the ARTEMIS-DELFI approach is tumor-and mutation-independent and can be applied even in settings where no tumor tissue is available for analysis. This approach does not rely on knowledge of specific mutations in a patient’s tumor, nor does it use a targeted panel. The ARTEMIS-DELFI method leverages the fragmentation features in addition to the repeat landscape across noncoding regions of the genome, hence deriving signal from a truly genome-wide picture of genomic, epigenomic and chromatin changes in cancer. Given the ease of obtaining plasma samples from patients, and the limited cfDNA used, all patients under evaluation were successfully analyzed.

While the CheckPAC and PACTO studies have provided substantial evidence that cfDNA analyses using WGMAF and ARTEMIS-DELFI correlate with patient outcome, the number of patients enrolled in both trials was limited. In addition, some serial blood sample time points did not correspond directly to dates of CT scans, and, therefore, they could not be directly compared. As is true for most studies of patients with pancreatic cancer, there were few responders in these trials, and, therefore, the number of patients with molecular responses represents a small subset of patients in these trials. In addition, cut points for determining molecular response in this study were not prespecified and based on quantiles of the observed responses in both the training and validation sets. Future studies in larger cohorts of patients with other types of cancer are needed to validate these methodologies.

In future clinical settings, these cfDNA-based methods could be used for monitoring patients over the course of treatment and making real-time clinical decisions about therapy regimens. Although both molecular approaches may be useful, the choice of which method to use may depend on the specific clinical application and sample availability. For patients with pancreatic cancer, methods for monitoring patients during therapy may be particularly important given the multitude of new avenues that are emerging for targeted and immune therapies.

## METHODS

### Sample collection

We examined 217 plasma samples for 40 of the 84 patients enrolled in a prospective study, CheckPAC (NCT02866383), at Copenhagen University Hospital–Herlev and Gentofte (*7*). In addition, we examined 205 plasma samples available for 40 of the 137 patients enrolled in a prospective study, PACTO (NCT02767557) (*33*), at Copenhagen University Hospital–Herlev and Gentofte. Material was examined as per the clinical study protocol. All patients provided informed consent, in agreement with the Helsinki Declaration. Patients also provided signed informed consent to be included in the Danish BIOPAC study “BIOmarkers in patients with PAncreatic Cancer (BIOPAC) - can they provide new information of the disease and improve diagnosis and prognosis of the patients?” (NCT03311776; www.herlevhospital.dk/BIOPAC). The BIOPAC study protocol has been approved by the Danish Ethics Committee (VEK, j.nr. KA-20060113) and the Danish Data Protection Agency (j.nr. 2006-41-6848, 2012-58-0004; HGH-2015-027; I-Suite j.nr. 03960; and PACTICUS P-2020-834). The study was conducted in accordance with the Declaration of Helsinki. At scheduled intervals throughout the course of treatment, patients had blood collected for research purposes. Patient tumor biopsies were performed before treatment initiation and 2 weeks after radiation treatment. At each sampling, two cancerous lesions were biopsied, and two cores were taken from each of the lesions. One core was formalin fixed and paraffin embedded and used for generation of slides with hematoxylin and eosin staining, while the other core was designated for molecular analyses. Half of the molecular analysis core was fresh frozen for genomic analyses. Frozen cores were kept at -80°C for long-term storage. Blood samples were collected in EDTA tubes and, within 3 hours after blood sampling, centrifuged at 2300*g* for 10 min at 4°C to separate plasma from buffy coat and red blood cell fractions. The EDTA plasma was stored in cryovials at −80°C until analysis. One milliliter of buffy coat was removed from each sample and stored in a cryovial at −80°C. Before analysis, the plasma fraction was thawed and spun at high speed of 18,000 *g* for 10 min to precipitate any remaining solid fraction. Liquid plasma fraction was aliquoted into cryovials and kept at −80°C for long-term storage.

### Tumor and buffy coat sample processing

Forty of the 43 patients in the CheckPAC study had plasma samples available. Frozen biopsies of the patients’ cancers that were available and had sufficient pathologic cellularity were selected for sequencing. Preference was given to pretreatment biopsies from a site that would not receive radiation, although in cases of insufficient material or cellularity, on-treatment and irradiated samples were sequenced. We evaluated tumor and matched buffy coat for the subset of 34 patients. Sample preparation and bioinformatic analyses of tumor and buffy coat samples were performed as previously described (*50*). Briefly, DNA was extracted from matched frozen tumor tissue and buffy coat samples using the Qiagen DNA FFPE Tissue Kit or Qiagen DNA Blood Mini Kit (Qiagen GmbH). Genomic libraries were prepared and whole-genome sequenced on Illumina NovaSeq 6000 S4 flow cells with 100-bp reads (table S3).

### Plasma sample processing

Circulating cfDNA was isolated from frozen plasma using Qiagen QIAamp Circulating Nucleic Acids Kit (Qiagen GmbH) from a median of 2.6 ml (range: 1 to 4 ml) of plasma, which was eluted in 52 μl of ribonuclease-free water with 0.04% sodium azide (Qiagen GmbH.). DNA was quantified, and quality was assessed from 1 μl of DNA eluate using Agilent 2100 Bioanalyzer automated electrophoresis platform, using DNA 1000 assay and chip. This yielded a median of 58.2 ng of DNA from each sample (range: 1.4 to 2600 ng). DNA was stored in LoBind tubes (Eppendorf AG) at −20°C. Concentration and quality of cfDNA were assessed using the Bioanalyzer 2100 (Agilent Technologies).

Samples were organized for next-generation sequencing (NGS) library preparation in batches of 11 to 26 samples. All sample time points for each patient were batched together, and each batch contained one nucleosome-digested control of healthy DNA (tables S5 and S6).

NGS cfDNA libraries were prepared for WGS using a maximum of 15 ng of cfDNA (tables S5 and S6). In brief, genomic libraries were prepared using the NEBNext DNA Library Prep Kit for Illumina (New England Biolab) with four main modifications to the manufacturer’s guidelines: (i) The library purification steps followed the on-bead AMPure XP (Beckman Coulter) approach to minimize sample loss during elution and tube transfer steps; (ii) NEBNext End Repair, A-tailing, and adapter ligation enzyme and buffer volumes were adjusted as appropriate to accommodate on-bead AMPure XP purification; (iii) Illumina dual index adapters were used in the ligation reaction; and (iv) cfDNA libraries were amplified with Phusion Hot Start Polymerase. All samples underwent a four-cycle PCR amplification after the DNA ligation step. Samples were run on Illumina NovaSeq 6000 S4 flow cells with 2 × 100–bp read length at a coverage of 2 to 3×. Sequencing data were output into FASTQ file format.

For 10 patients in the CheckPAC trial, targeted sequencing was performed using the targeted error correction sequencing (TEC-Seq) approach to evaluate 64 well-known cancer driver genes (*51*). This method used the PlasmaSELECT 64 gene panel from Personal Genome Diagnostics and is based on targeted capture and deep sequencing (>30,000×) of DNA fragments and enables identification of tumor-specific alterations in ctDNA while distinguishing these from amplification and sequencing artifacts, germline changes, or alterations related to blood cell proliferation that may be present in cfDNA.

### Preprocessing of sequencing data

Before alignment, adapter sequences were filtered from reads using fastp version 0.20.0 (*52*). Sequence reads were aligned against the hg19 human reference genome using Bowtie2 version 2.3.0 (*53*), and duplicate reads were removed using Sambamba 0.6.8 (*54*) (plasma) or Samtools version 1.18 (*55*) (tumor and buffy coat).

### Selection of sample time points

Patient baseline time points were selected as the plasma draw that occurred before treatment initiation. Follow-up evaluation time point was selected as the second blood draw that occurred after treatment initiation, with a median of 55 days (fig. S2B and tables S5 and S6).

### Estimation of cfDNA MAF from WGS

Somatic single-base substitutions were identified in tumor tissue from a joint analysis of tumor and matched normal WGS data using Strelka2 version 2.9.0 (*56*). Variant Call Format (VCF) files were filtered for candidate variants passing all Strelka2 filters (VCF FILTER field set to PASS). We further filtered variant calls to require (i) ≥3 mutant tier 1 reads from the tumor, (ii) ≥10% MAF of tier 1 reads from the tumor (iii) ≥30× coverage of tier 1 reads from the matched normal, (iv) zero mutant tier 1 reads from the matched normal, and (v) ≤5% MAF of tier 2 reads from the matched normal. In addition, we removed any candidate variants that were in the gnomAD database (version 3.0) with an allele frequency >1 in 10,000, or if the variant did not pass gnomAD quality filters.

For each genomic position harboring a somatic mutation in the tumor after filtering (variant positions), we determined the base call of overlapping reads in cfDNA sequencing data using the pileup method in pysam version 0.16.0.1. Only proper read pairs with the highest mapping quality (MAPQ) of 42 were considered and reads flagged as PCR or optical duplicates as well as read pairs where either mate contained ≥5 uncalled (*N*) bases were excluded. Furthermore, we required that bases from both read 1 and read 2 of a read pair overlapped a given variant position with Phred score ≥30, contained either the reference or the mutant allele, and agreed on the base call. A single MAF value was computed for each cfDNA sample as the number of observed mutant bases divided by the total number of bases analyzed after filtering, where counts were summed across all variant positions, genome wide.

Validation of WGMAF was performed using targeted sequencing TEC-Seq approach from PGDx (*51*) of a subset of patients in CheckPAC trial. For all patients with *KRAS* mutations, we used the *KRAS* MAF values for comparison. In the case of patient CGPLPA223, where no *KRAS* mutation was detected, we selected the highest observed MAF in *CTNNB1*.

### Fragmentation analysis

Fragmentation features were calculated as previously described (*27*). Briefly, each aligned pair was converted to a genomic interval representing the sequenced DNA fragment using bedtools 2.29.0 (*57*). Only reads with a MAPQ score of at least 30 were retained. Read pairs were further filtered if overlapping the Duke Excluded Regions blacklist (https://genome.ucsc.edu/cgi-bin/hgTrackUi?db=hg19&g=wgEncodeMapability). To capture large-scale epigenetic differences in fragmentation across the genome estimable from low-coverage WGS, we tiled the hg19 reference genome into nonoverlapping 5-Mb bins. Bins with an average GC base content<0.3 and an average mappability <0.9 were excluded, leaving 473 bins spanning approximately 2.4 Gb of the genome. GC correction was performed independently for short (<150 bp) and long (≥150 bp) cfDNA fragments using an external panel of 20 individuals without cancer sequenced on a NovaSeq to generate a target distribution. *Z* scores representing arm gains/losses were calculated for autosomal chromosome arms from the total number of aligned fragments and then centered and scaled by the mean representation and standard deviation from an external reference set as previously described (*27*).

### Chromatin structure analysis

PDAC A/B compartments were generated from TCGA methylation data according to (*47*). We used reference lymphoblastoid cell line Hi-C data from https://github.com/Jfortin1/HiC_AB_Compartments, as described previously (*47*). The two reference tracks were compared to identify informative 100-kb bins, defined as bins where the chromatin domain differed between the two reference tracks or the magnitude difference in eigenvalues corresponded to a *z* score greater than 1.96 or less than −1.96 (*P* = 0.05) across all eigenvalue differences. The median fragmentation profile for six CheckPAC samples with high estimated tumor fraction by WGMAF and 10 randomly selected individuals without cancer was calculated. This information was used to extract an estimated median PDAC component in the plasma weighted by the WGMAF value of the individual plasma samples.

### ARTEMIS-DELFI score calculation

We computed kmer repeat landscapes for all samples as previously described (*30*). We selected 786 features with more than 1000 kmers per million aligned reads and centered and scaled kmer counts within each sample across repeat families. We also computed GC corrected coverage in 559 1-Mb bins with high density of epigenetic marks. Using data from a prior multi-cancer cohort (LUCAS) (*27*), we trained an ARTEMIS ensemble model to differentiate patients with cancer from those without. The ARTEMIS ensemble was composed of six penalized logistic regression (PLR) models with repeat landscapes as features (one PLR for each of five repeat families and for the epigenetic profile) that together generated the ARTEMIS score. We further trained two DELFI fragmentation feature models—a PLR on the principal components capturing 95% of variance of the 473 bin-level fragmentation ratios, and a PLR on the chromosomal arm *z* scores. We ensembled the two scores from these models with the ARTEMIS score to train the ARTEMIS-DELFI model. The locked models trained on the LUCAS cohort (*27*) were used to generate ARTEMIS-DELFI scores for the CheckPAC and PACTO cohorts. DELFI-TF analyses were performed as described in van’t Erve *et al.* (*31*).

### Tissue copy number analyses

Copy number analyses were performed on the tumor and matched buffy coat BAM files using FACETS version 0.5.0: allele-specific copy number and clonal heterogeneity analysis tool for high-throughput DNA sequencing (*45*). Arm level copy number odds ratios were calculated as the median across FACETS-generated segments for each chromosomal arm. FACETS was also used to estimate tumor cellularity.

Copy number analyses were performed on the plasma BAM files using ichorCNA from the Broad Institute (*20*). Arm level copy number odds ratios were calculated as the median across ichorCNA generated segments for each chromosomal arm. IchorCNA was also used to estimate plasma tumor fraction.

### Cox proportional hazards

We conducted Cox proportional hazards analyses to assess whether the values from WGMAF and ARTEMIS-DELFI independently predict OS. The multivariate models for WGMAF and ARTEMIS-DELFI were adjusted for treatment and clinical covariates previously reported in Johansen *et al.* (*58*): ECOG PS, MGPS, age (greater than 65 years), sex, weight loss (greater than or equal to 5%), and bilirubin (greater than 25 μM).

In (*7*), data from 84 patients were used to build a multivariate model to predict OS. WGS data were available for 40 patients in CheckPAC and 40 in PACTO. For analyses incorporating both baseline and on-treatment WGS data (ARTEMIS-DELFI or DELFI TF), we included a total of 29 patients. Because of the additional requirement of tumor samples for WGMAF, this analysis had a total of 17 patients. In PACTO, baseline and on-treatment WGS data were available for 32 samples.

In (*7*), the authors performed univariate analyses to identify covariates for multivariate survival analysis by selecting covariates that were statistically significant with a type I error of 5%. We replicated the multivariate analysis from (*58*)—a follow-up analysis to (*7*)—using a smaller sample size and omitting WGS data to observe changes in effect sizes and significance. Our analysis defined progression-free survival and OS from the start of treatment rather than the date of randomization; we verified that this change did not affect effect sizes or significance. After restricting the sample size to patients with WGS data, none of the covariates of (*58*) remained statistically significant. In (*58*), MGPS, ECOG PS, bilirubin, and weight loss ≥5% were significant covariates. In CheckPAC, none of the patients with WGS data had high bilirubin, so this variable was excluded. However, bilirubin was retained in PACTO because of the presence of patients with elevated levels. Data for ECOG PS and weight loss ≥5% were unavailable for PACTO patients, so these variables were excluded in that study. The patients selected for multivariate analysis in CheckPAC were further narrowed to those with both baseline and on-treatment WGS data, and the findings remained unchanged: None of the clinical covariates were statistically significant.

We then conducted univariate analyses on the baseline and on-treatment values of DELFI TF, WGMAF, and ARTEMIS-DELFI in CheckPAC patients. Only on-treatment scores were significant, with HR >1. Baseline scores were not significant for any, with HR <1, except for ARTEMIS-DELFI, where HR >1. Subsequently, we fit multivariate models incorporating both baseline and on-treatment values of DELFI TF, WGMAF, and ARTEMIS-DELFI. For DELFI TF and WGMAF, two model versions were tested: baseline with on-treatment values and baseline with the change in values (defined as the difference between on-treatment and baseline values). Note that effect sizes and significance for other covariates do not change in these versions, only the interpretation of the effects of baseline and on-treatment or baseline and change in values changes. In the model version with baseline and change in values, baseline values had HR > 1. This is because keeping a fixed “change in values” implies raising on-treatment value when baseline value is raised. Since ARTEMIS-DELFI represents a cancer risk score ranging from 0 to 1, interpreting the difference between on-treatment and baseline values was deemed inappropriate, so this model version was excluded. To maintain consistent covariates across models, we opted to include baseline and on-treatment values in multivariate analyses. We applied a percentage change criterion to DELFI TF and WGMAF in CheckPAC, but these models did not validate in PACTO. We suspect that although DELFI TF and WGMAF values predict OS, study-related differences (e.g., previous therapies and treatment types) preclude the generalization of change-point cutoffs from one study to another. Therefore, we used continuous values for ARTEMIS-DELFI and standardized WGMAF in multivariate analysis. Treatment arm was added as a covariate to the models. CheckPAC and PACTO each had two arms: In CheckPAC, arm 1 received radiotherapy with nivolumab, and arm 2 received radiotherapy with nivolumab and ipilimumab; in PACTO, arm 1 received chemotherapy with nab-paclitaxel, and arm 2 received chemotherapy with nab-paclitaxel and tocilizumab.

### Statistical analysis

All statistical analyses were performed using R 4.4.0. Statistical significance of Kaplan-Meier survival curves was assessed using the log-rank test. HRs for OS and PFS clinical end points were calculated from univariate and multivariate Cox proportional hazards regression. CIs for WGMAF, ARTEMIS-DELFI, and ichorCNA were calculated on the logit scale and then transformed back to the nominal scale with the inverse logit. CIs and *t* tests for CA19-9 were evaluated on the log_10_ scale.
