# Supplementary material for: Genome-wide analyses of cell-free DNA for therapeutic monitoring of patients with pancreatic cancer
Source: Sci Adv. 2025 May 21;11(21):eads5002. doi: 10.1126/sciadv.ads5002 (PMC12094228; doi:10.1126/sciadv.ads5002)
Supplement: Supplementary file 1 — Figs. S1 to S24 Legends for tables S1 to S6 [file sciadv.ads5002_sm.pdf]

Science Advances
AAAS

Supplementary Materials for

Genome-wide analyses of cell-free DNA for therapeutic monitoring of patients with pancreatic cancer

Carolyn Hruban *et al.*

Corresponding author: Zachariah H. Foda, foda@jhmi.edu; Jillian Phallen, jphalle2@jhmi.edu; Victor E. Velculescu, velculescu@jhmi.edu

Sci. Adv. **11**, eads5002 (2025)
DOI: 10.1126/sciadv.ads5002

The PDF file includes:

Figs. S1 to S24
Legends for tables S1 to S6

Other Supplementary Material for this manuscript includes the following:

Tables S1 to S6

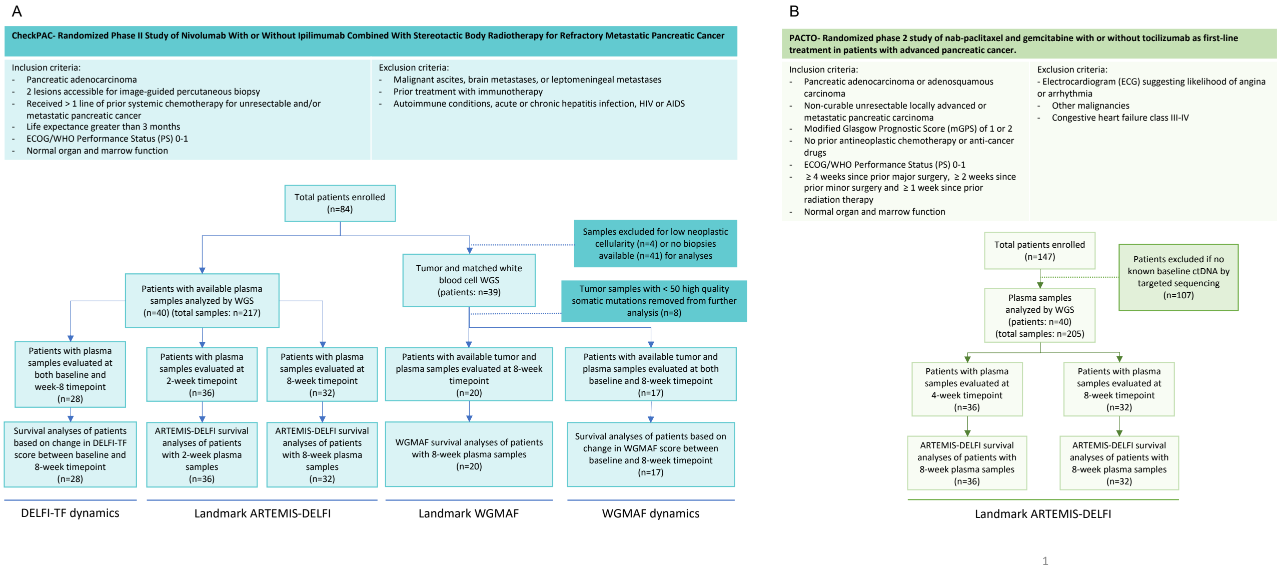

fig. S1. Flowchart of sample selection for WGMAF and ARTEMIS-DELFI analyses. Charts showing criteria for inclusion and exclusion criteria as well as analyses performed using patient samples from the (A) CheckPAC and (B) PACTO trials.

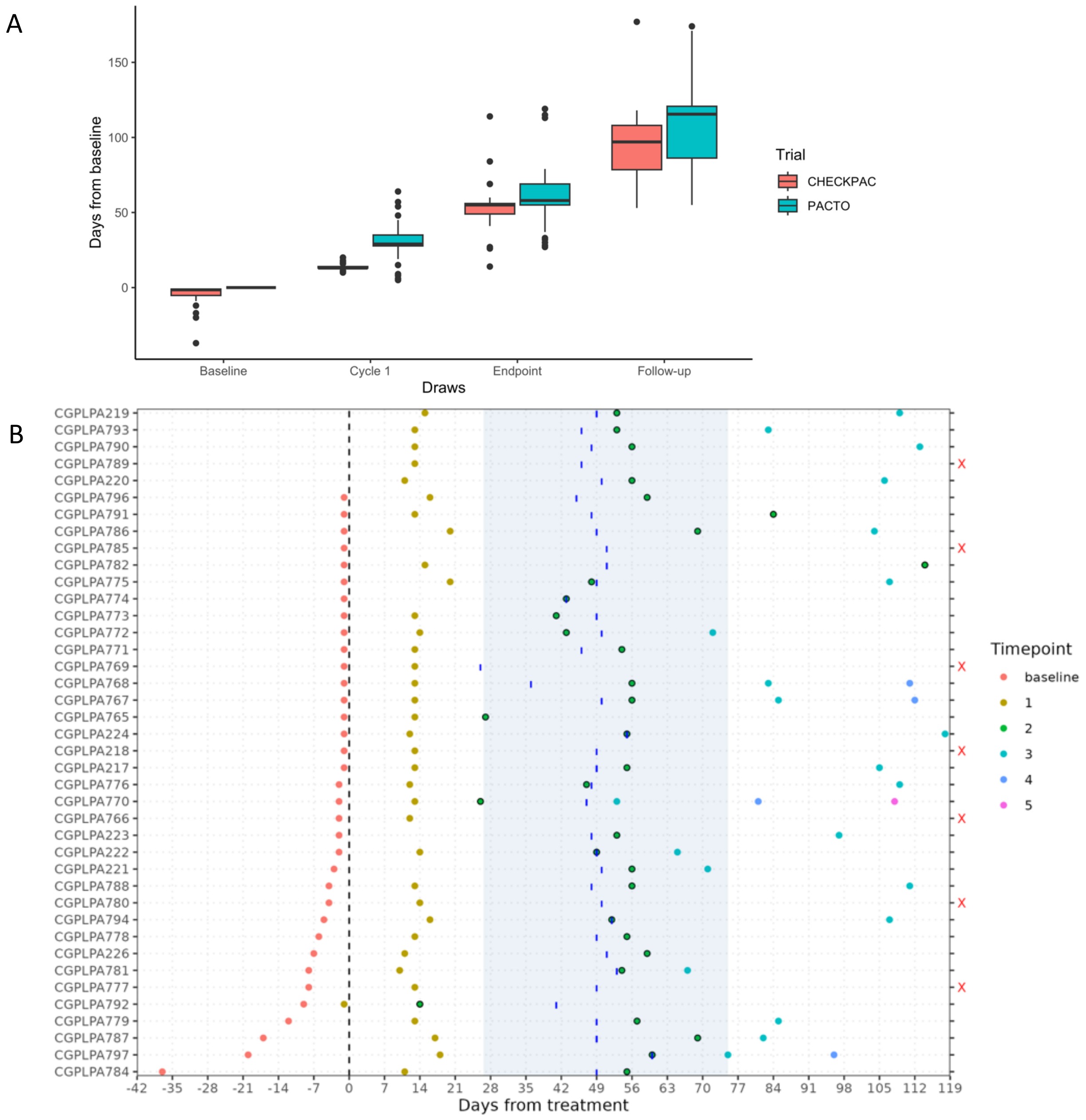

fig. S2. Selection of post-treatment liquid biopsy timepoints for molecular analyses. (A) Time distribution of each blood draw in CheckPAC and PACTO studies. (B) Plot showing blood draw dates and CT timepoints selected for analyses for CheckPAC trial. Blue lines indicate the date of CT. Red "X" marks indicate patients dropped due to missing blood draw. The highlighted blue region indicates the 0.05-0.95 quantile range of timepoint selection. The analyzed second follow-up timepoints are outlined in black. In the legend, the colors indicate whether the timepoint is from baseline or from a follow-up blood draw (1-5).

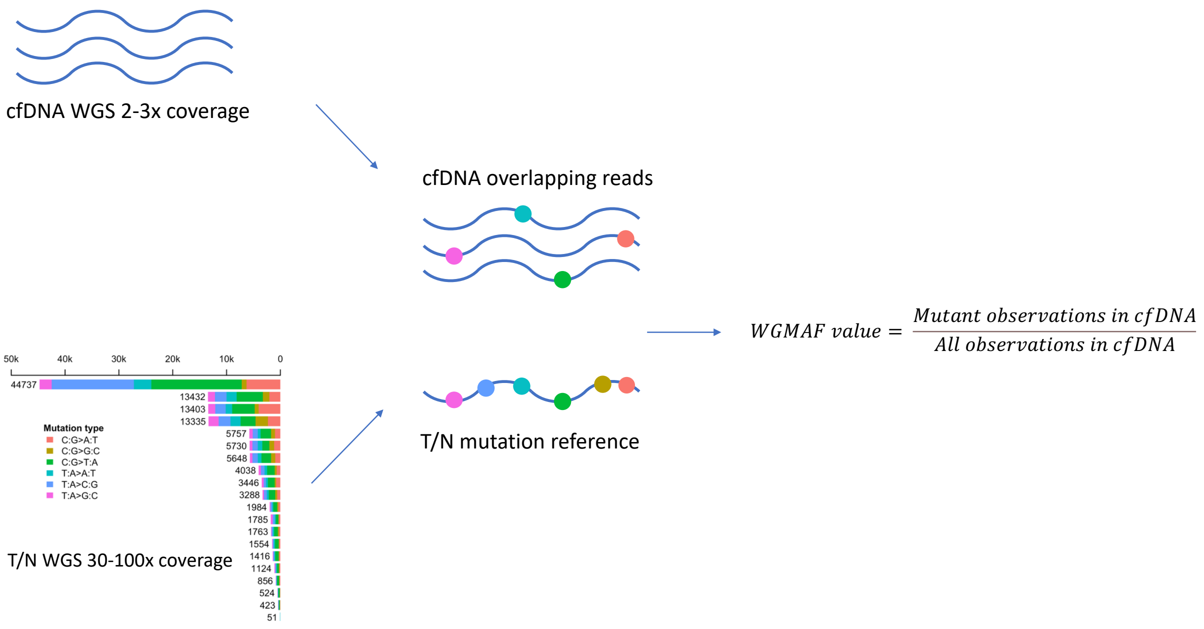

fig. S3. Overview of WGMAF method. For each patient, the tumor and matched normal tissue WGS analyses resulted in a set of somatic mutations for further evaluation. Analyses of the positions of these mutations in matching cfDNA using WGS allows determination of the WGMAF score which is defined as the sum of mutated observations across all observations at these positions. The WGMAF score provides an estimate of the MAF for each plasma sample.

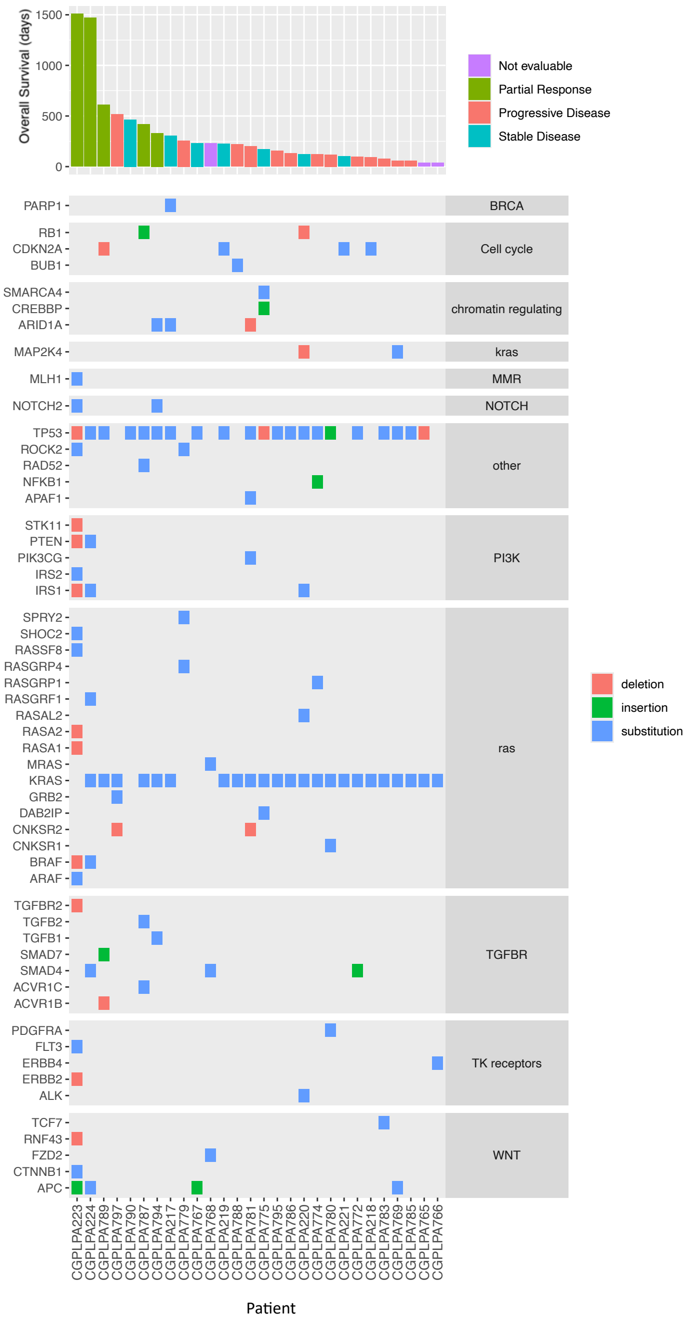

fig. S4. Genome-wide mutational landscape of patients with pancreatic cancer in CheckPAC study. Overall survival is colored at the top, by clinical partial response (PR), stable disease (SD), and progressive disease (PD). Single base substitution mutations are indicated along vertical axis, sorted by pathway. Patients are listed along the horizontal axis. Mutations are colored according to mutation type.

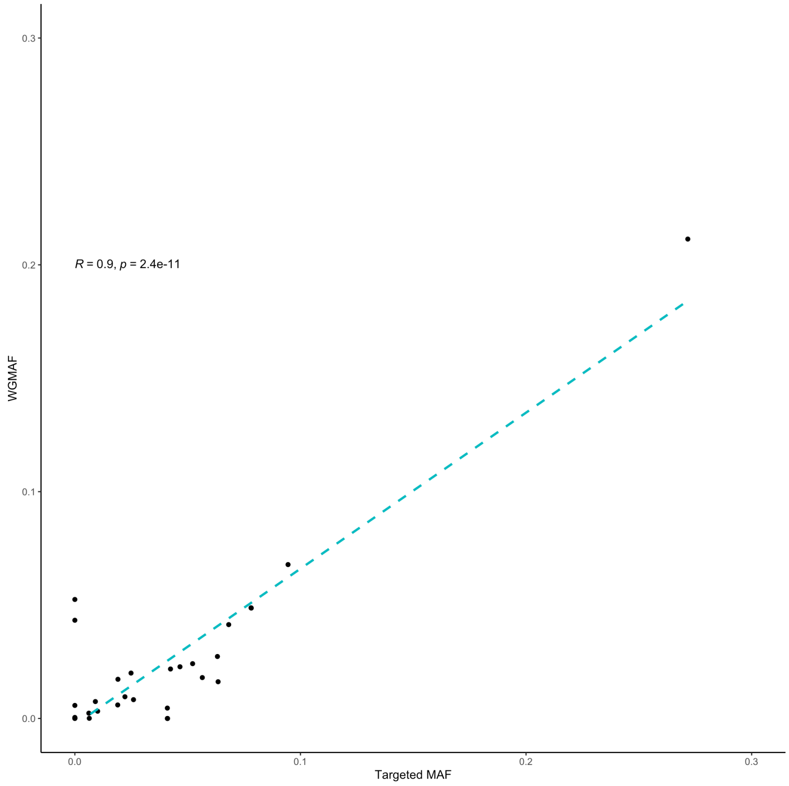

fig. S5. WGMAF values correlate with targeted MAF analyses. WGMAF compared to targeted MAF across plasma samples assessed by targeted deep sequencing in subset of patients. Targeted MAF represents the fraction of mutant to wildtype *KRAS* sequences in cfDNA; for patients with no *KRAS* mutation the targeted MAF represents the highest fraction of mutant to wildtype sequences observed in that sample.

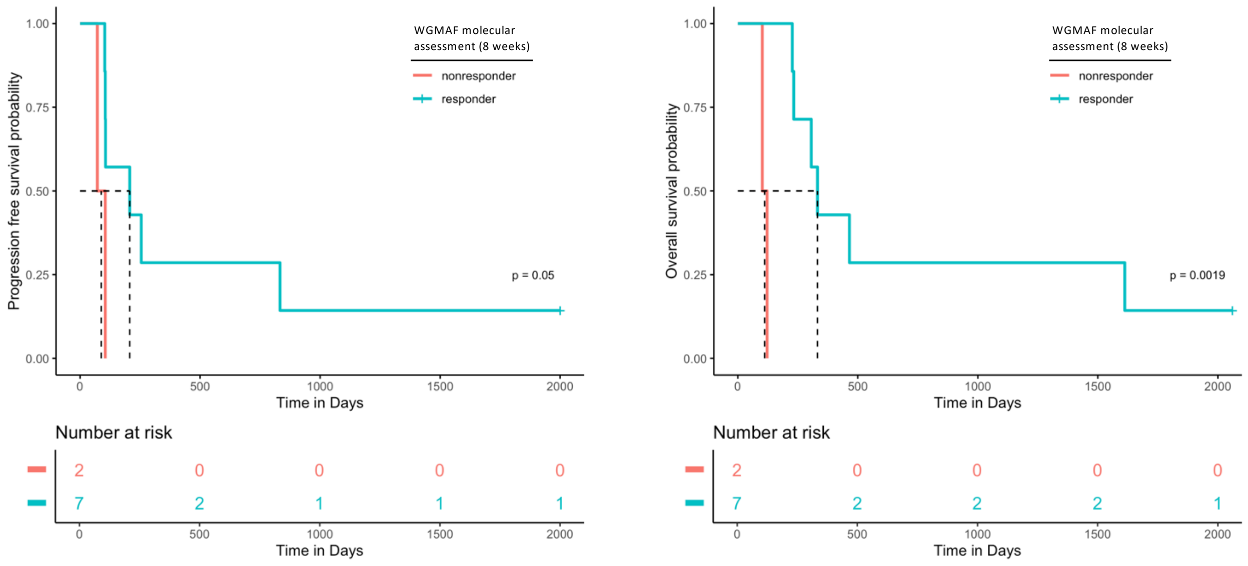

fig. S6. WGMAF stratifies progression-free and overall survival for patients in the CheckPAC trial with stable disease. Kaplan-Meier curves of progression-free survival probability and overall survival probability based on landmark WGMAF values at the 8-week timepoint. Analyses were performed for patients having stable disease at the first follow-up CT scan.

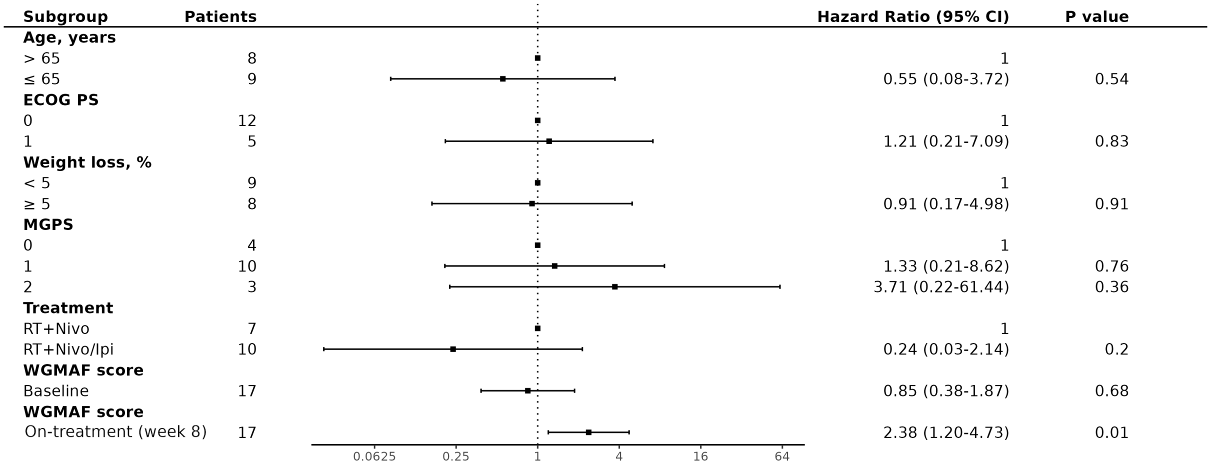

fig. S7. Multivariate hazard analyses demonstrate on-treatment WGMAF values as independent predictors of overall survival for patients in the CheckPAC trial. Multivariate Cox proportional hazard analyses were generated for each molecular method and fit to overall survival adjusting for clinical subgroups. Subgroups previously shown to be significant in univariate analyses (*7*) were included in the multivariate analysis. WGMAF scores were assessed at baseline and at 8-week timepoint for patients in the CheckPAC study.

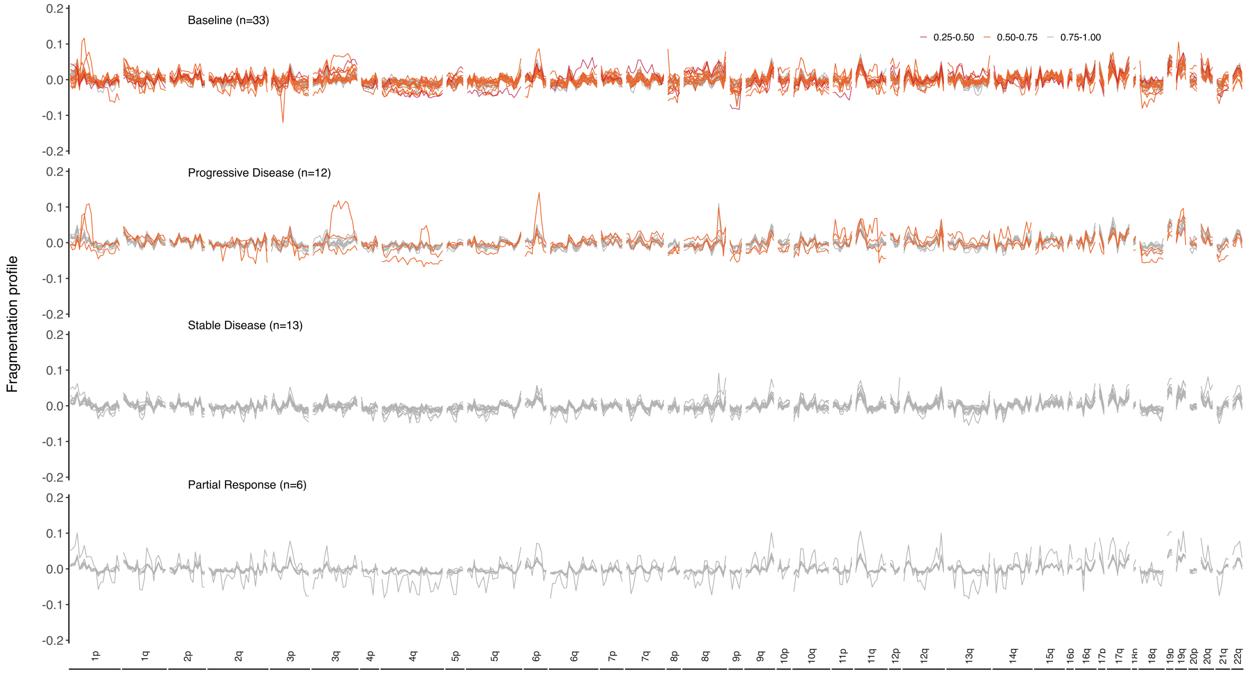

fig. S8. Fragmentation patterns for patients in CheckPAC trial with partial response or stable disease are more closely correlated to healthy plasma. cfDNA fragmentation profiles from CheckPAC patients are shown as short (100 - 150 bp) to long (151 - 220 bp) ratios of fragment sizes in 473 bins 5-Mb in size across the genome. Profiles are shown for all patients with plasma samples at baseline and at follow-up for each of the clinical RECIST 1.1 response categories. Each profile is colored by correlation to the median profile of healthy reference samples

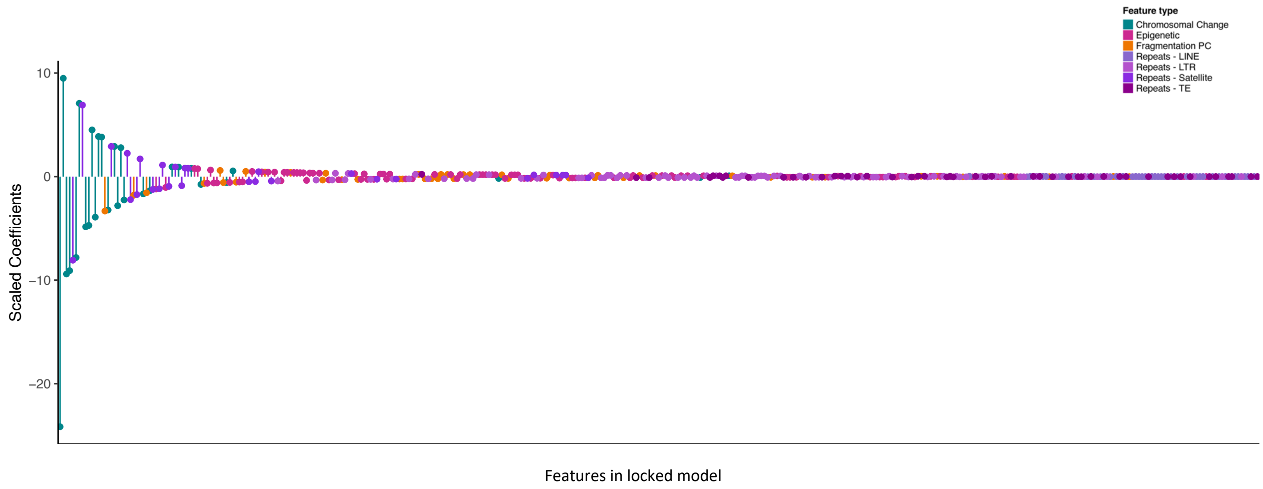

fig. S9. Feature importance for locked ARTEMIS-DELFI machine learning model. Scaled coefficients are indicated along y axis for each feature along x axis. Features are colored by feature type.

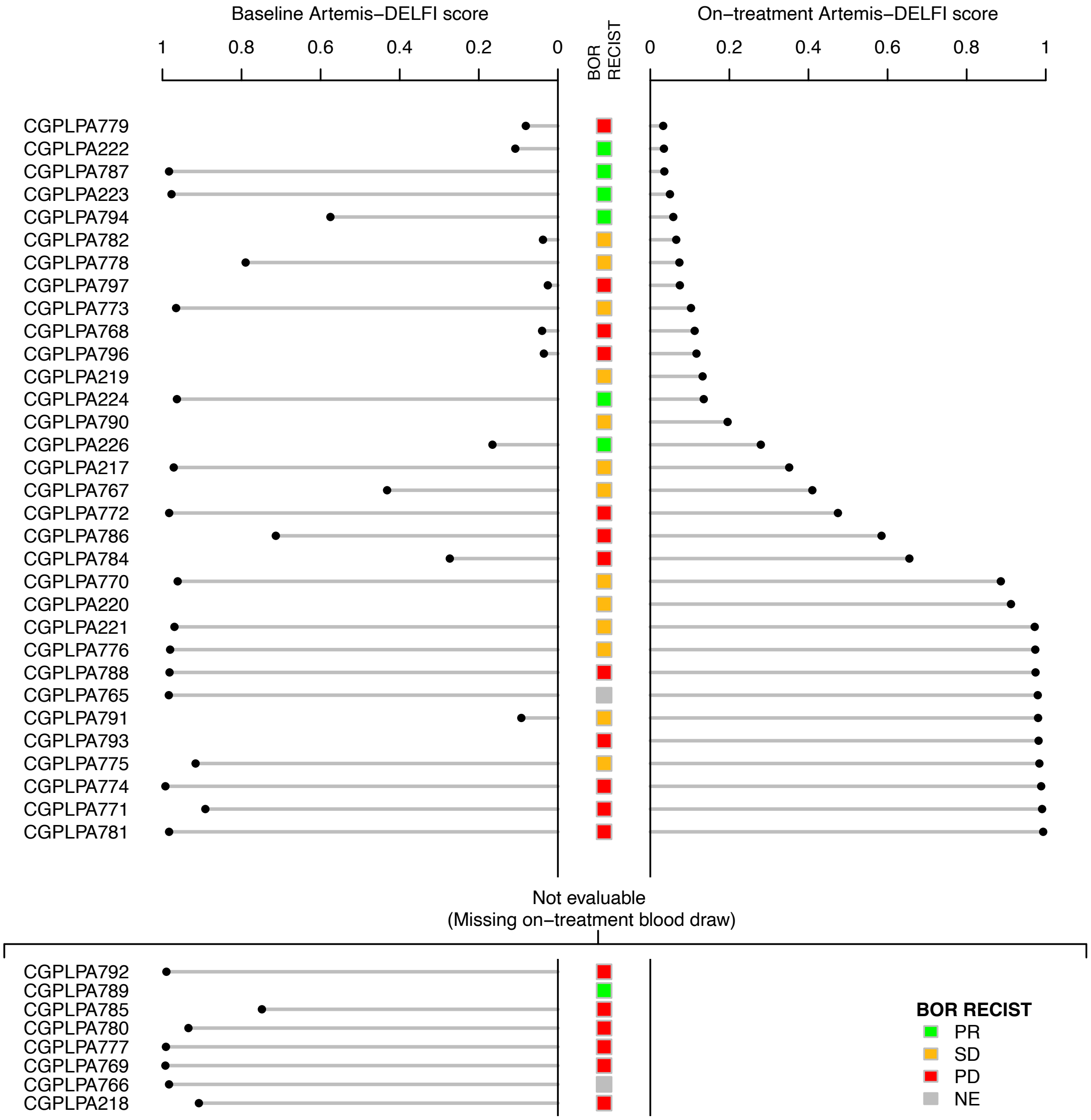

fig. S10. ARTEMIS-DELFI scores and best overall response in CheckPAC trial. Patients are listed along vertical axis, separated into those that are evaluable by ARTEMIS-DELFI method at the follow-up timepoint. Baseline ARTEMIS-DELFI scores are plotted on the left side of the plot, with follow-up ARTEMIS-DELFI scores on the right side of the plot. BOR RECIST 1.1 is shown in central panel.

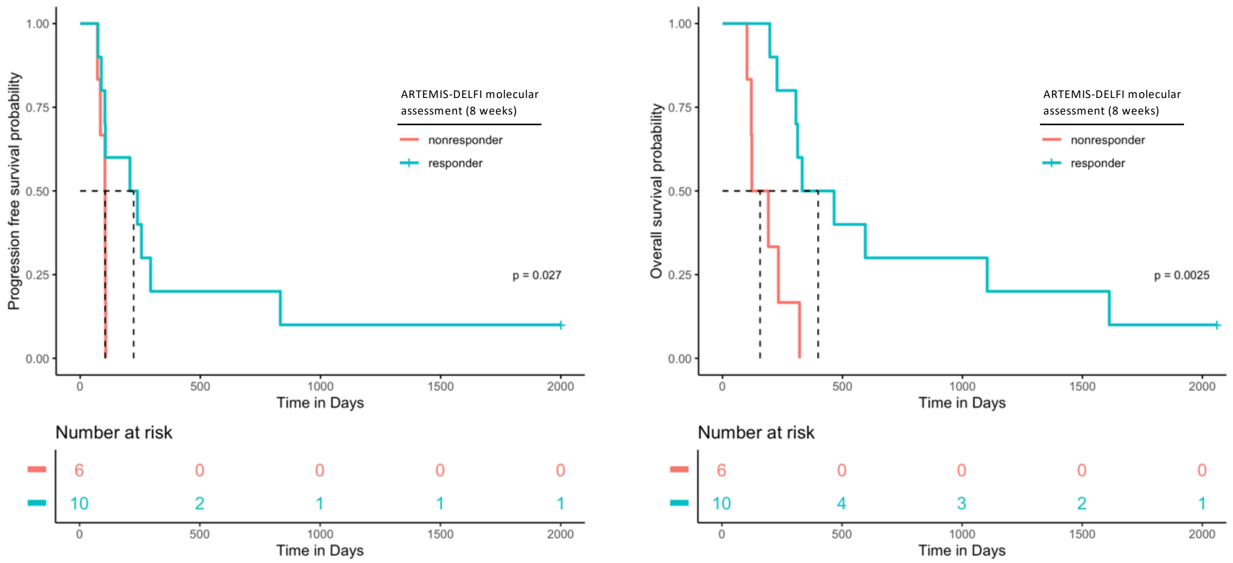

fig. S11. ARTEMIS-DELFI stratifies progression-free and overall survival for patients in CheckPAC trial with stable disease. Kaplan-Meier curves of progression-free survival probability and overall survival probability based on landmark ARTEMIS-DELFI score at 8-week timepoint, limited to patients assessed as having stable disease at the first follow-up CT scan.

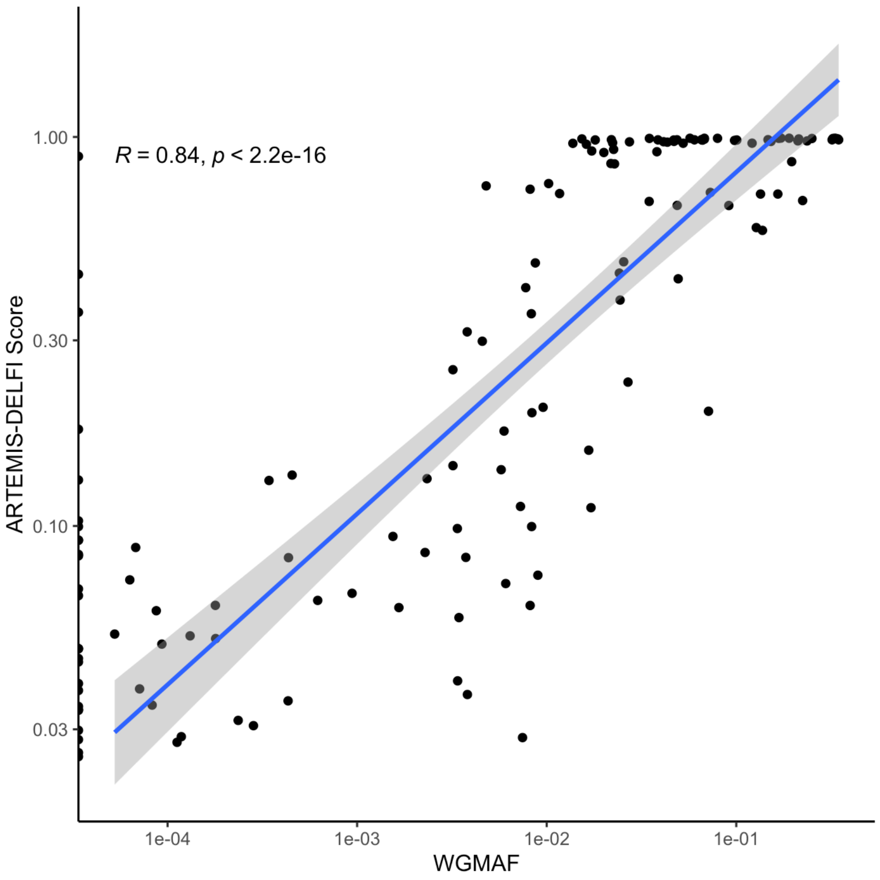

fig. S12. ARTEMIS-DELFI scores and WGMAF values are closely correlated. ARTEMIS-DELFI scores and WGMAF values are plotted against each other with trendline indicated.

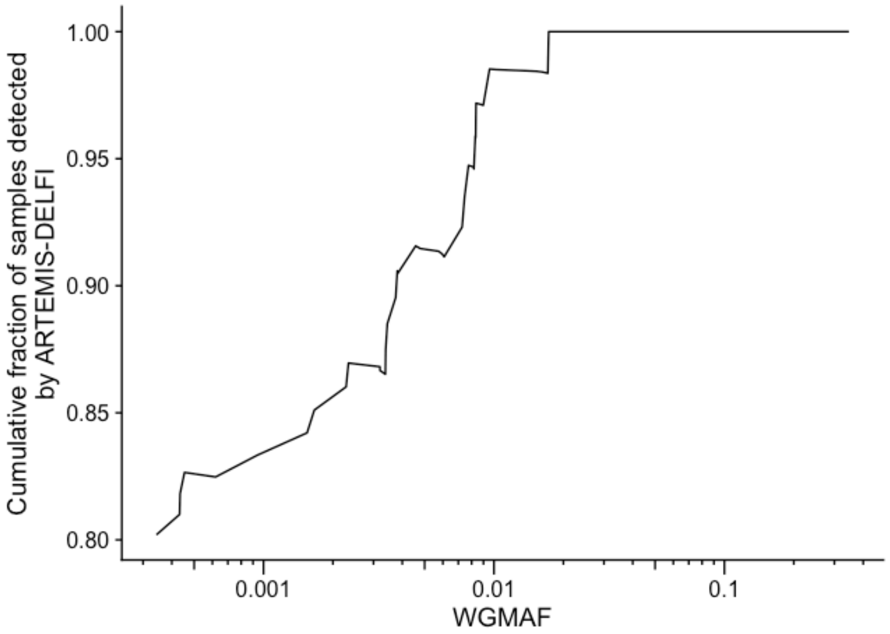

fig. S13. Fraction of samples detected using ARTEMIS-DELFI at different ctDNA concentrations. For different ctDNA concentrations as determined by WGMAF, the fraction of samples detected by ARTEMIS-DELFI is indicated from .8-1 on the y axis.

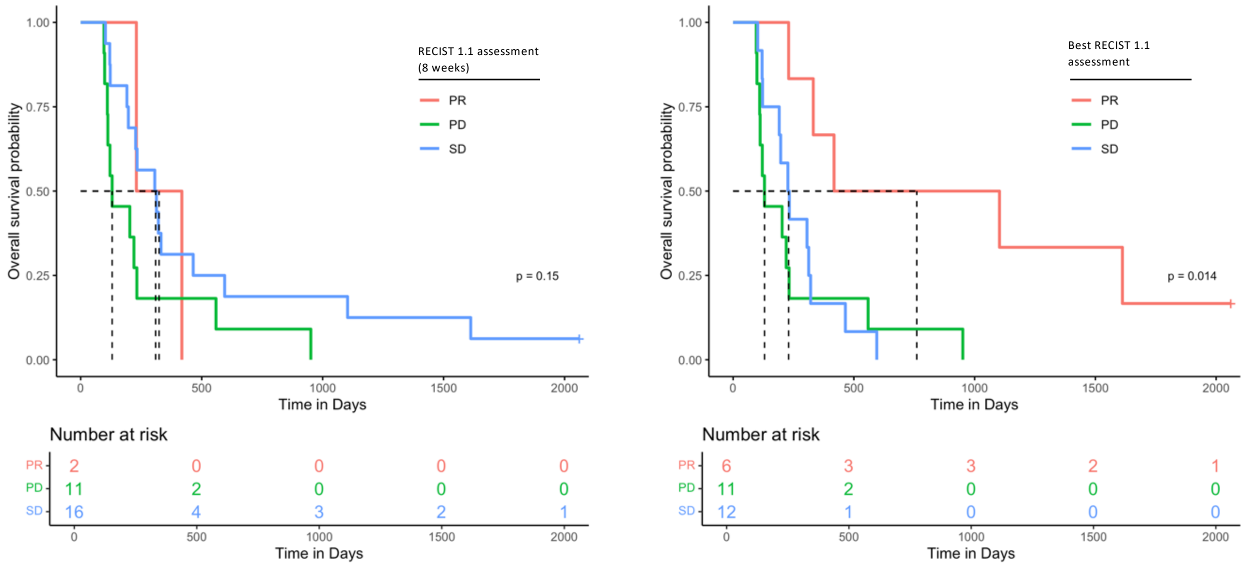

fig. S14. Survival analyses for RECIST scoring at first follow-up scan and for BOR RECIST in CheckPAC trial. Kaplan-Meier curves of overall survival probability based on first RECIST follow-up scan, and clinical BOR. Patients are Separated into partial response, stable disease, and progressive disease.

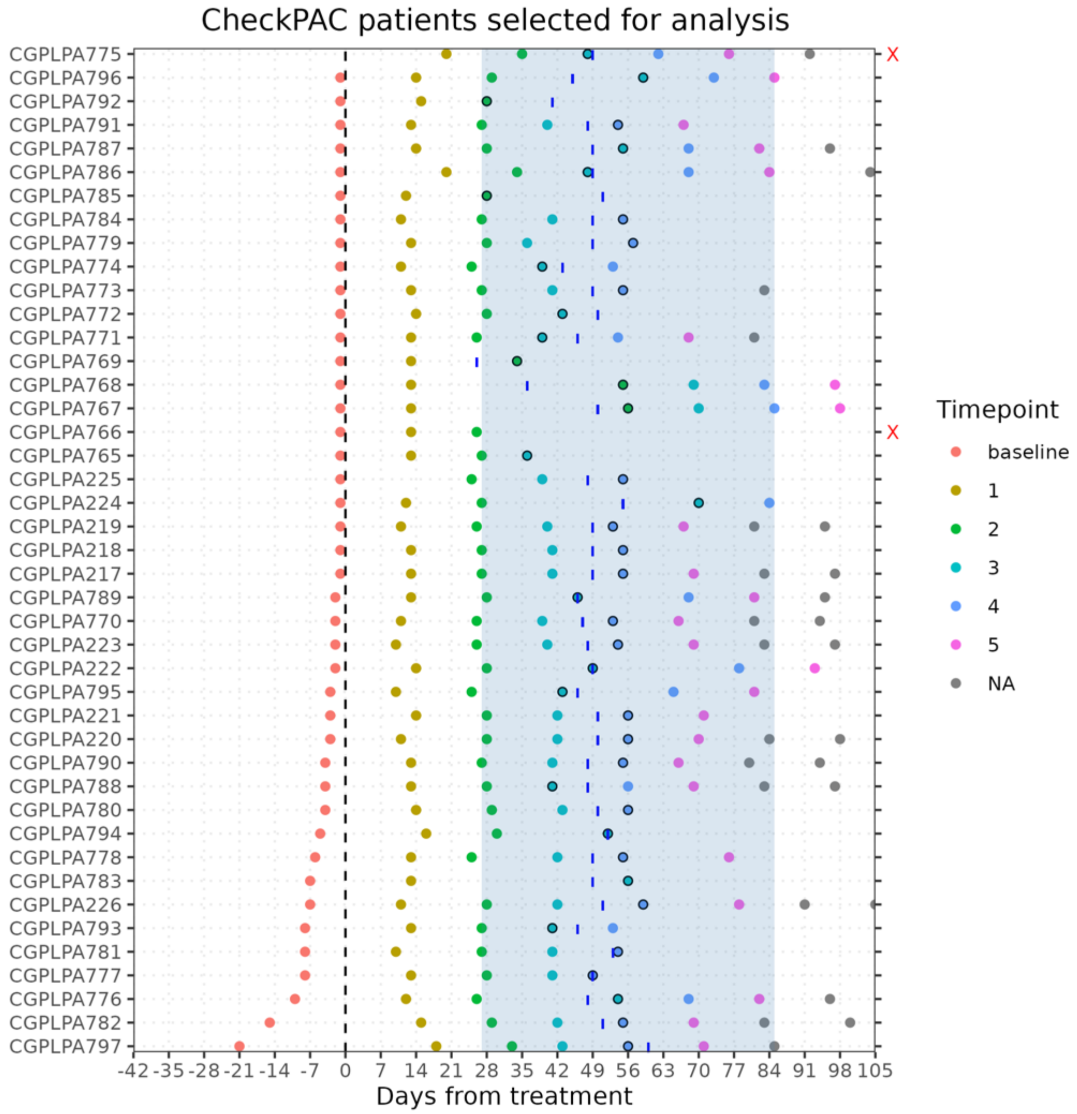

fig. S15. Timepoints selected for CA19-9 measurements and CT imaging for patients in CheckPAC trial. (A) Blue lines indicate the date of CT. Red "X" marks indicate patients dropped due to missing blood draw. The highlighted blue region indicates the 0.05-0.95 quantile range of timepoint selection. The analyzed fourth follow-up CA19-9 timepoints are outlined in black. In the legend, the colors indicate whether the timepoint is from baseline or from a follow-up blood draw (1-5).

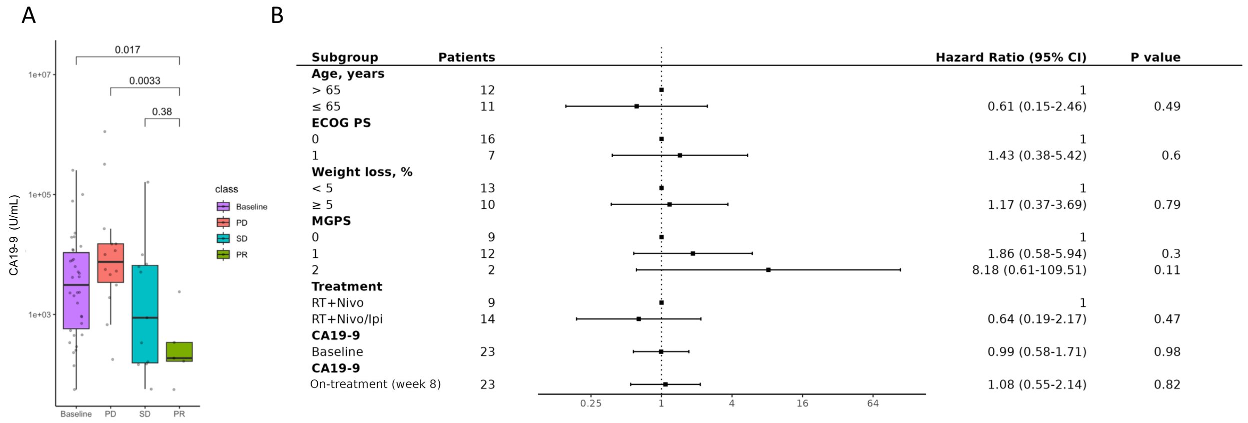

fig. S16. Landmark CA19-9 levels show limited correlation with clinical response for patients in CheckPAC trial. (A) Boxplot showing CA19-9 concentration at baseline and at follow-up time points for patients with PD, SD, and PR. Wilcoxon P values are indicated above each comparison. (B) Multivariate cox proportional hazard analyses were generated for each molecular method and fit to overall survival adjusting for clinical subgroups. Subgroups previously shown to be significant in univariate analyses (*7*) were included in the multivariate analysis. Hazard ratios and P values are indicated on the right for individuals in the CheckPAC study, after excluding those who do not secrete CA19-9.

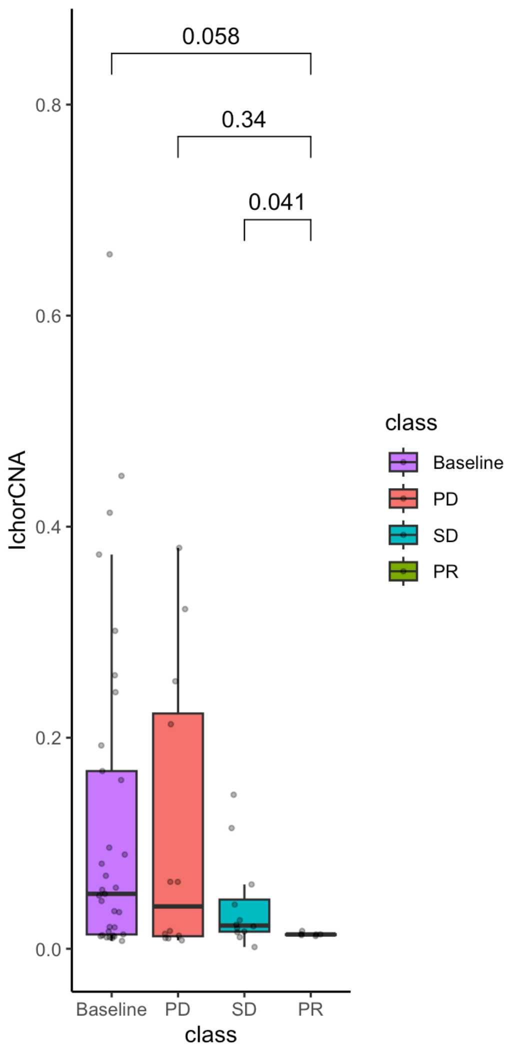

fig. S17. ichorCNA scores show limited correlation with clinical response. Boxplot showing ichorCNA tumor fraction, at baseline and at follow-up time points for patients with PD, SD, and PR. Wilcoxon P-values are indicated above each comparison.

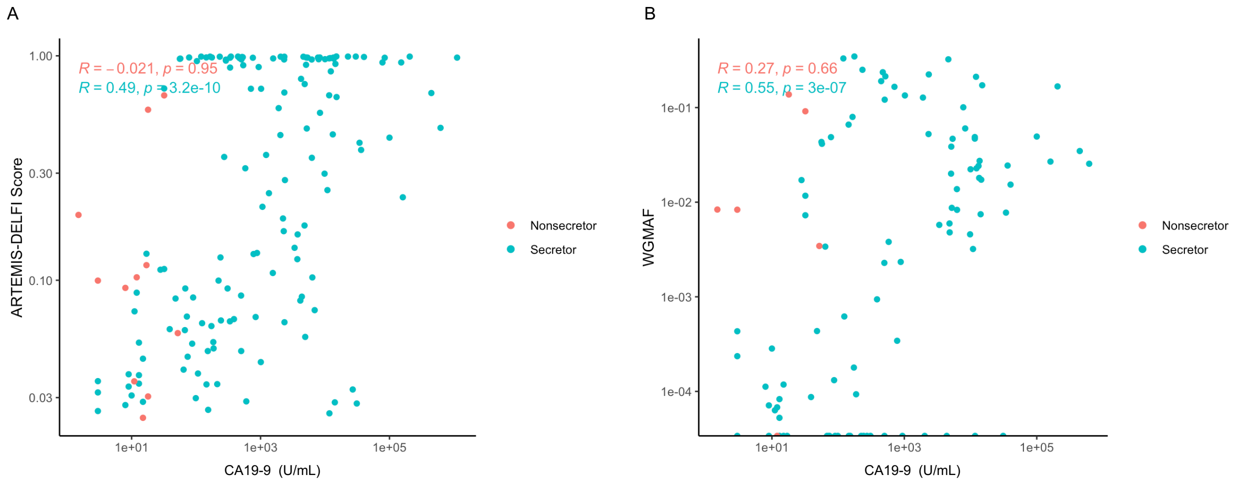

fig. S18. CA19-9 concentrations were associated ARTEMIS-DELFI and WGMAF values for patients in CheckPAC trial who were secretors but not for non-secretors. Scatter plots showing correlation of ARTEMIS-DELFI scores with CA19-9 concentrations on the left, and WGMAF values with CA19-9 concentrations on the right. Patient samples for non-secretors are colored in red, while secretors are colored in blue.

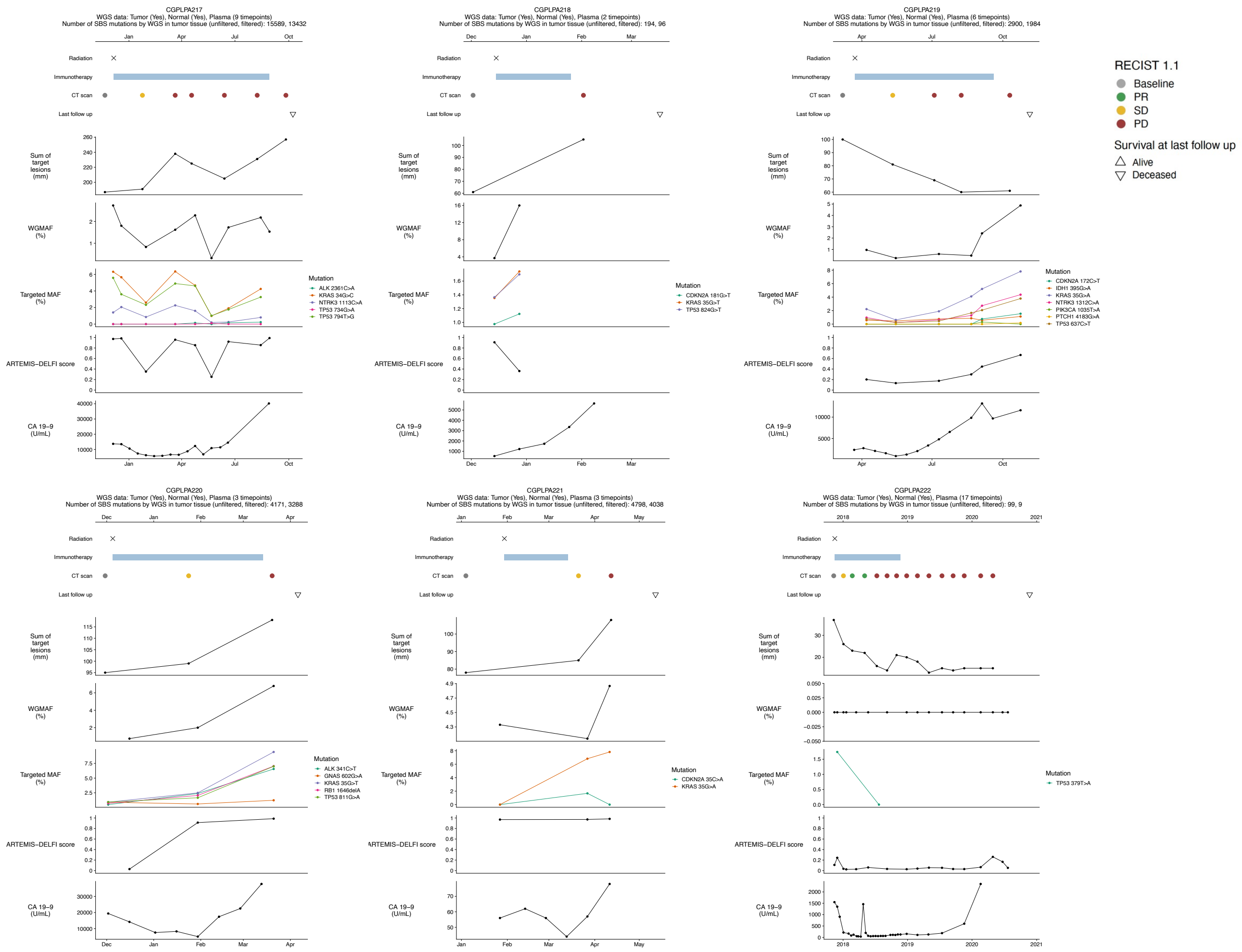

CGPLPA217
WGS data: Tumor (Yes), Normal (Yes), Plasma (9 timepoints)
Number of SBS mutations by WGS in tumor tissue (unfiltered, filtered): 15589, 13432
CGPLPA218
WGS data: Tumor (Yes), Normal (Yes), Plasma (2 timepoints)
Number of SBS mutations by WGS in tumor tissue (unfiltered, filtered): 194, 96
CGPLPA219
WGS data: Tumor (Yes), Normal (Yes), Plasma (6 timepoints)
Number of SBS mutations by WGS in tumor tissue (unfiltered, filtered): 2900, 1984
CGPLPA220
WGS data: Tumor (Yes), Normal (Yes), Plasma (3 timepoints)
Number of SBS mutations by WGS in tumor tissue (unfiltered, filtered): 4171, 3288
CGPLPA221
WGS data: Tumor (Yes), Normal (Yes), Plasma (3 timepoints)
Number of SBS mutations by WGS in tumor tissue (unfiltered, filtered): 4798, 4038
CGPLPA222
WGS data: Tumor (Yes), Normal (Yes), Plasma (17 timepoints)
Number of SBS mutations by WGS in tumor tissue (unfiltered, filtered): 99, 9
Radiation
Immunotherapy
CT scan
Last follow up
Sum of target lesions (mm)
WGMAF (%)
Targeted MAF (%)
ARTEMIS–DELFI score
CA 19–9 (U/mL)
Mutation
ALK 2361C>A
KRAS 34G>C
NTRK3 1113C>A
TP53 734G>A
TP53 794T>G
CDKN2A 181G>T
KRAS 35G>T
TP53 824G>T
CDKN2A 172C>T
IDH1 395G>A
KRAS 35G>A
NTRK3 1312C>A
PIK3CA 1035T>A
PTCH1 4183G>A
TP53 637C>T
ALK 341C>T
GNAS 602G>A
KRAS 35G>T
RB1 1646delA
TP53 811G>A
CDKN2A 35C>A
KRAS 35G>A
TP53 379T>A
RECIST 1.1
Baseline
PR
SD
PD
Survival at last follow up
Alive
Deceased

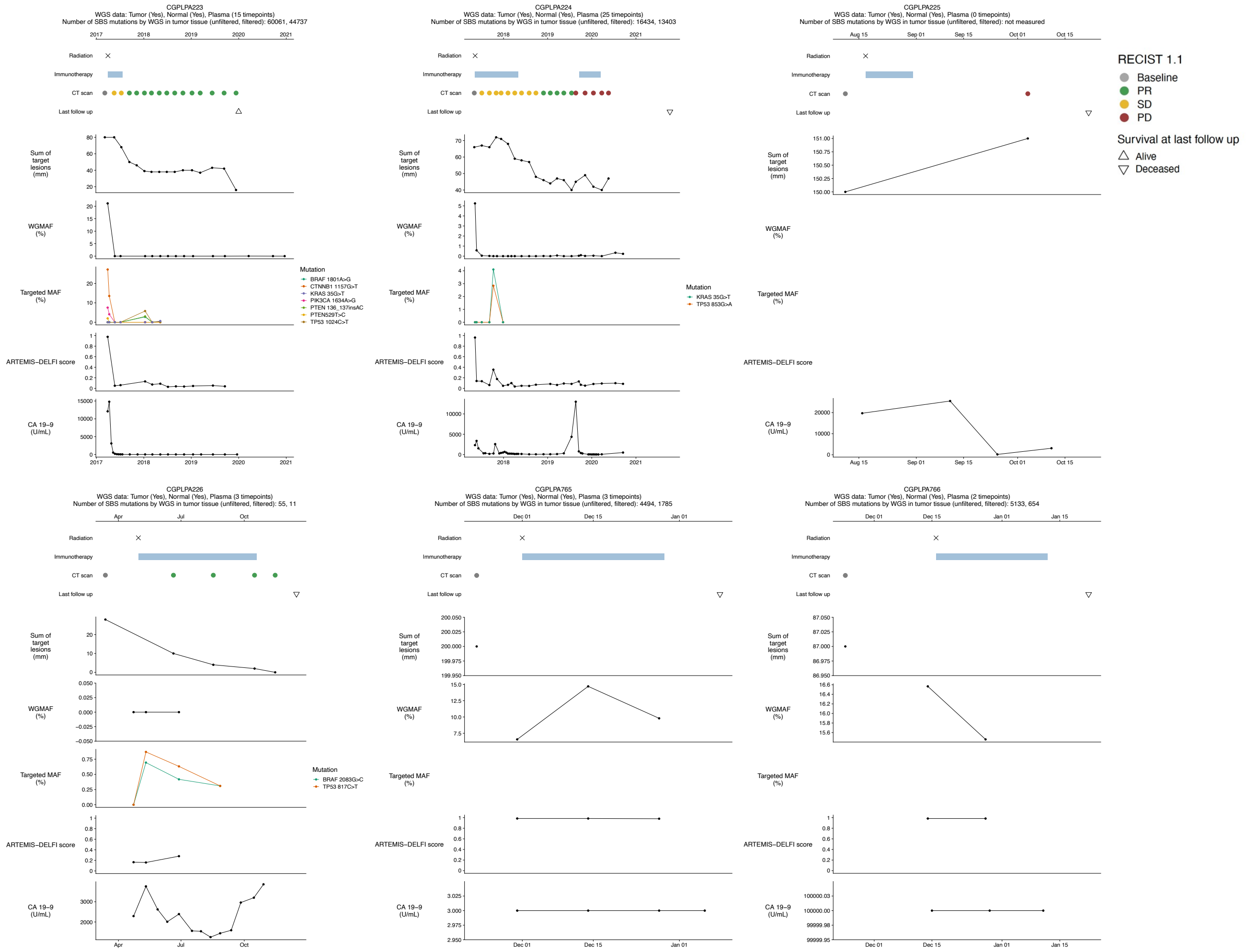
CGPLPA223
WGS data: Tumor (Yes), Normal (Yes), Plasma (15 timepoints)
Number of SBS mutations by WGS in tumor tissue (unfiltered, filtered): 60061, 44737
CGPLPA224
WGS data: Tumor (Yes), Normal (Yes), Plasma (25 timepoints)
Number of SBS mutations by WGS in tumor tissue (unfiltered, filtered): 16434, 13403
CGPLPA225
WGS data: Tumor (Yes), Normal (Yes), Plasma (0 timepoints)
Number of SBS mutations by WGS in tumor tissue (unfiltered, filtered): not measured
CGPLPA226
WGS data: Tumor (Yes), Normal (Yes), Plasma (3 timepoints)
Number of SBS mutations by WGS in tumor tissue (unfiltered, filtered): 55, 11
CGPLPA765
WGS data: Tumor (Yes), Normal (Yes), Plasma (3 timepoints)
Number of SBS mutations by WGS in tumor tissue (unfiltered, filtered): 4494, 1785
CGPLPA766
WGS data: Tumor (Yes), Normal (Yes), Plasma (2 timepoints)
Number of SBS mutations by WGS in tumor tissue (unfiltered, filtered): 5133, 654
Radiation
Immunotherapy
CT scan
Last follow up
Sum of target lesions (mm)
WGMAF (%)
Targeted MAF (%)
ARTEMIS–DELFI score
CA 19–9 (U/mL)
Mutation
BRAF 1801A>G
CTNNB1 1157G>T
KRAS 35G>T
PIK3CA 1634A>G
PTEN 136_137insAC
PTEN529T>C
TP53 1024C>T
KRAS 35G>T
TP53 853G>A
BRAF 2083G>C
TP53 817C>T
RECIST 1.1
Baseline
PR
SD
PD
Survival at last follow up
Alive
Deceased

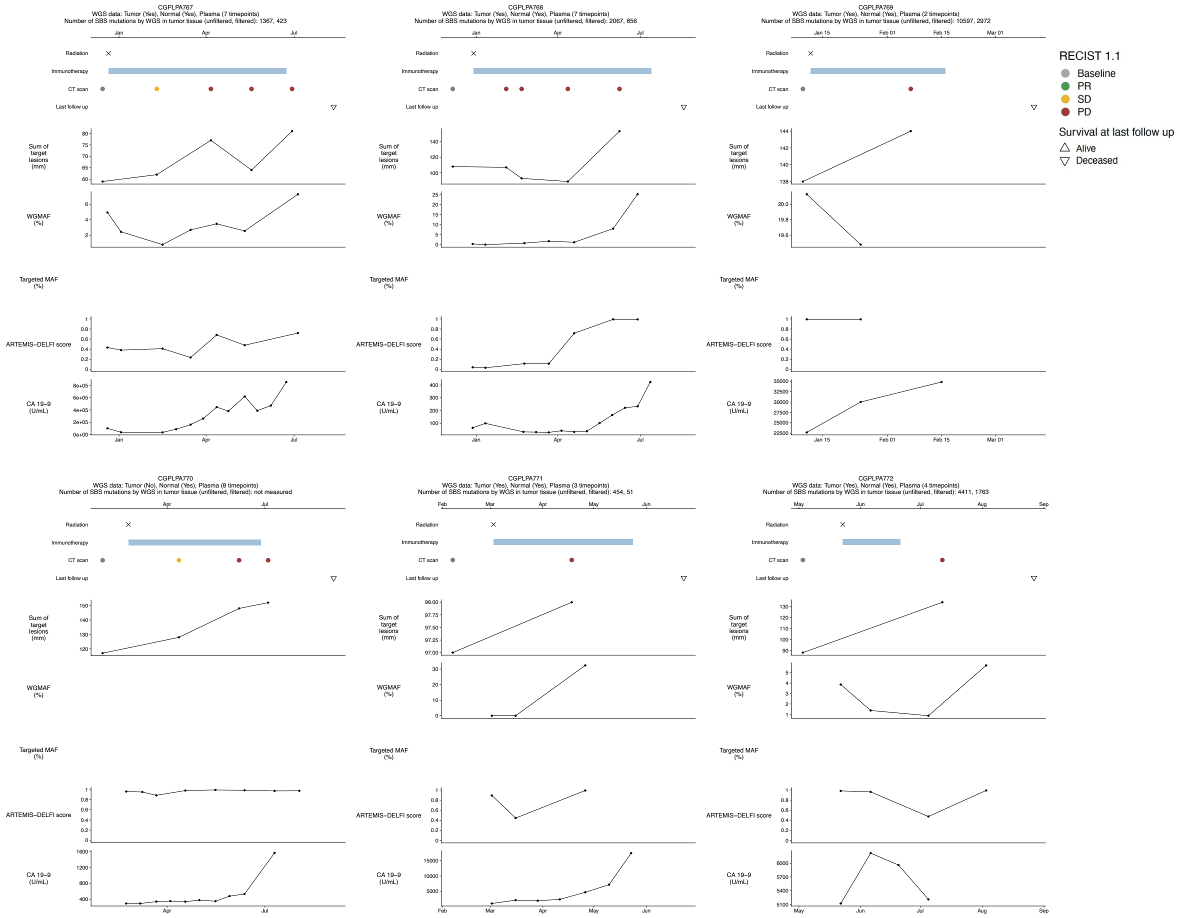

CGPLPA767
WGS data: Tumor (Yes), Normal (Yes), Plasma (7 timepoints)
Number of SBS mutations by WGS in tumor tissue (unfiltered, filtered): 1367, 423
CGPLPA768
WGS data: Tumor (Yes), Normal (Yes), Plasma (7 timepoints)
Number of SBS mutations by WGS in tumor tissue (unfiltered, filtered): 2067, 856
CGPLPA769
WGS data: Tumor (Yes), Normal (Yes), Plasma (2 timepoints)
Number of SBS mutations by WGS in tumor tissue (unfiltered, filtered): 10597, 2972
CGPLPA770
WGS data: Tumor (No), Normal (Yes), Plasma (8 timepoints)
Number of SBS mutations by WGS in tumor tissue (unfiltered, filtered): not measured
CGPLPA771
WGS data: Tumor (Yes), Normal (Yes), Plasma (3 timepoints)
Number of SBS mutations by WGS in tumor tissue (unfiltered, filtered): 454, 51
CGPLPA772
WGS data: Tumor (Yes), Normal (Yes), Plasma (4 timepoints)
Number of SBS mutations by WGS in tumor tissue (unfiltered, filtered): 4411, 1763
Radiation
Immunotherapy
CT scan
Last follow up
Sum of target lesions (mm)
WGMAF (%)
Targeted MAF (%)
ARTEMIS–DELFI score
CA 19–9 (U/mL)
RECIST 1.1
Baseline
PR
SD
PD
Survival at last follow up
Alive
Deceased

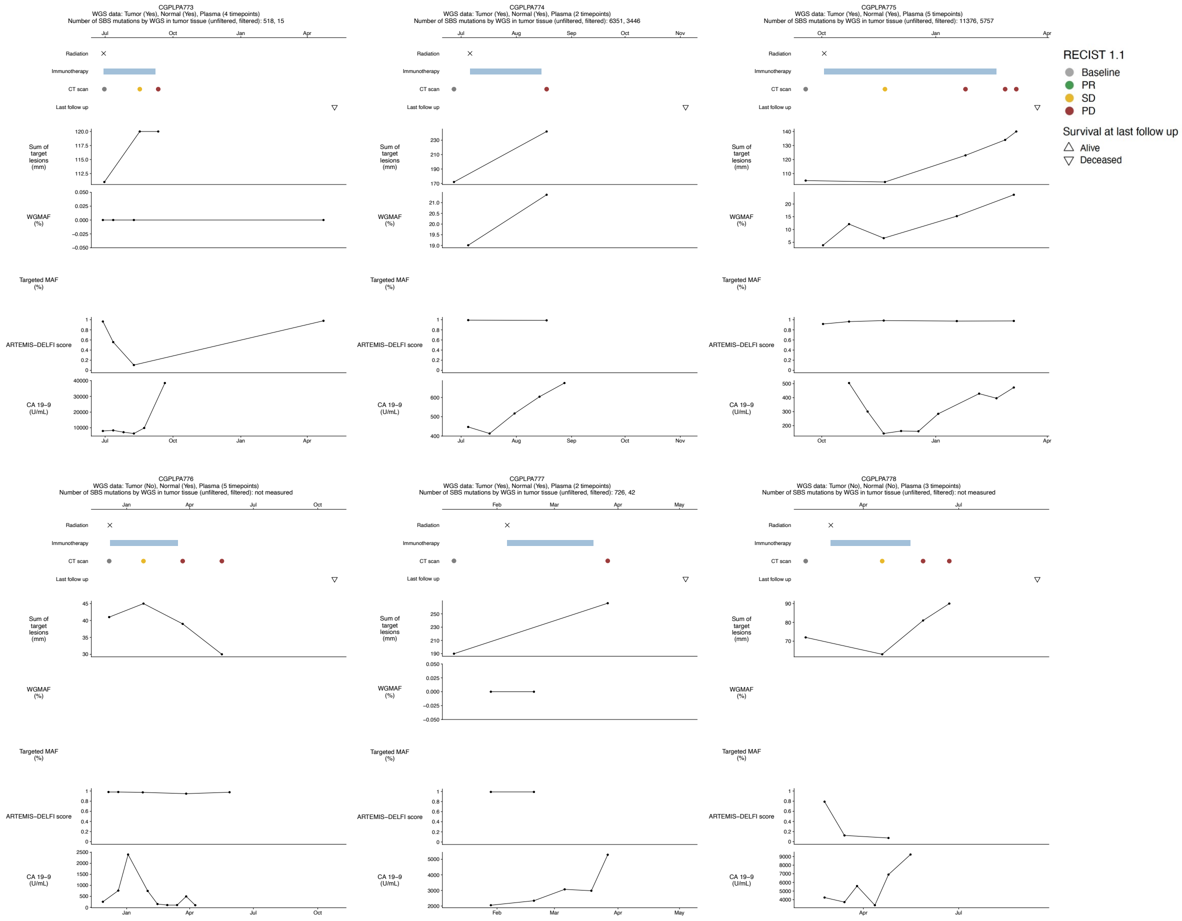
CGPLPA773
WGS data: Tumor (Yes), Normal (Yes), Plasma (4 timepoints)
Number of SBS mutations by WGS in tumor tissue (unfiltered, filtered): 518, 15
CGPLPA774
WGS data: Tumor (Yes), Normal (Yes), Plasma (2 timepoints)
Number of SBS mutations by WGS in tumor tissue (unfiltered, filtered): 6351, 3446
CGPLPA775
WGS data: Tumor (Yes), Normal (Yes), Plasma (5 timepoints)
Number of SBS mutations by WGS in tumor tissue (unfiltered, filtered): 11376, 5757
CGPLPA776
WGS data: Tumor (No), Normal (Yes), Plasma (5 timepoints)
Number of SBS mutations by WGS in tumor tissue (unfiltered, filtered): not measured
CGPLPA777
WGS data: Tumor (Yes), Normal (Yes), Plasma (2 timepoints)
Number of SBS mutations by WGS in tumor tissue (unfiltered, filtered): 726, 42
CGPLPA778
WGS data: Tumor (No), Normal (No), Plasma (3 timepoints)
Number of SBS mutations by WGS in tumor tissue (unfiltered, filtered): not measured
Radiation
Immunotherapy
CT scan
Last follow up
Sum of target lesions (mm)
WGMAF (%)
Targeted MAF (%)
ARTEMIS–DELFI score
CA 19–9 (U/mL)
RECIST 1.1
Baseline
PR
SD
PD
Survival at last follow up
Alive
Deceased

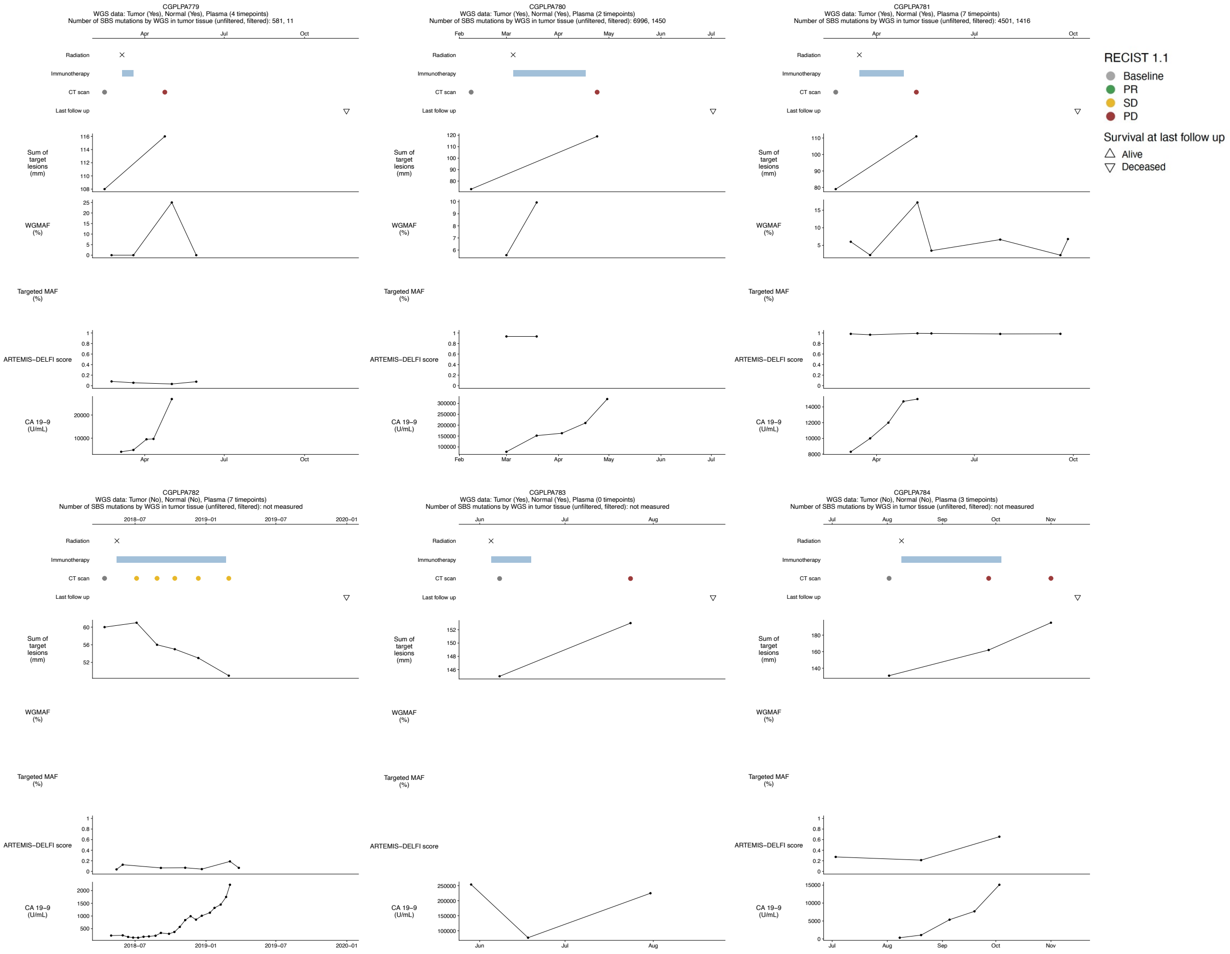
CGPLPA779
WGS data: Tumor (Yes), Normal (Yes), Plasma (4 timepoints)
Number of SBS mutations by WGS in tumor tissue (unfiltered, filtered): 581, 11
CGPLPA780
WGS data: Tumor (Yes), Normal (Yes), Plasma (2 timepoints)
Number of SBS mutations by WGS in tumor tissue (unfiltered, filtered): 6996, 1450
CGPLPA781
WGS data: Tumor (Yes), Normal (Yes), Plasma (7 timepoints)
Number of SBS mutations by WGS in tumor tissue (unfiltered, filtered): 4501, 1416
CGPLPA782
WGS data: Tumor (No), Normal (No), Plasma (7 timepoints)
Number of SBS mutations by WGS in tumor tissue (unfiltered, filtered): not measured
CGPLPA783
WGS data: Tumor (Yes), Normal (Yes), Plasma (0 timepoints)
Number of SBS mutations by WGS in tumor tissue (unfiltered, filtered): not measured
CGPLPA784
WGS data: Tumor (No), Normal (No), Plasma (3 timepoints)
Number of SBS mutations by WGS in tumor tissue (unfiltered, filtered): not measured
Radiation
Immunotherapy
CT scan
Last follow up
Sum of target lesions (mm)
WGMAF (%)
Targeted MAF (%)
ARTEMIS–DELFI score
CA 19–9 (U/mL)
RECIST 1.1
Baseline
PR
SD
PD
Survival at last follow up
Alive
Deceased

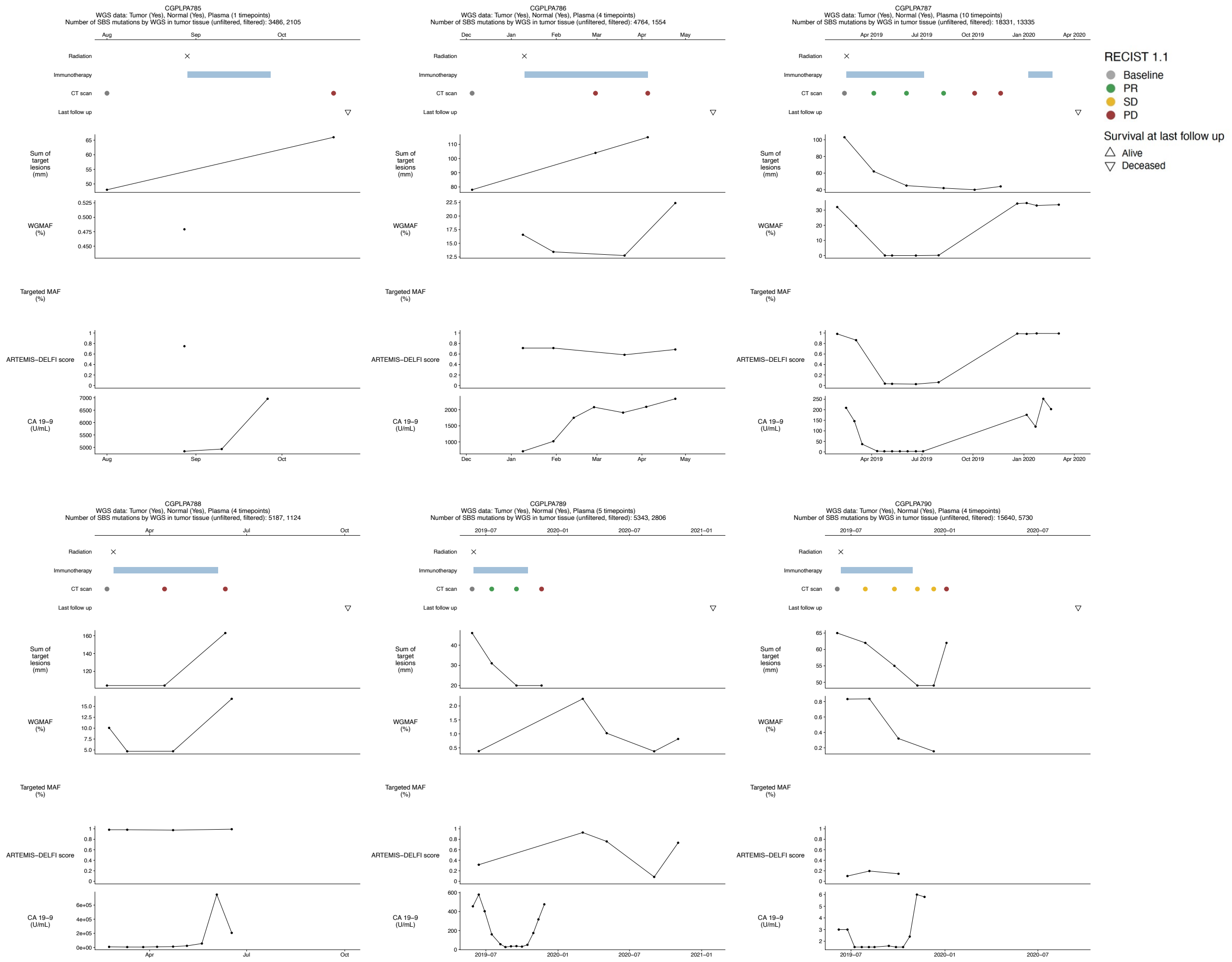
CGPLPA785
WGS data: Tumor (Yes), Normal (Yes), Plasma (1 timepoints)
Number of SBS mutations by WGS in tumor tissue (unfiltered, filtered): 3486, 2105
CGPLPA786
WGS data: Tumor (Yes), Normal (Yes), Plasma (4 timepoints)
Number of SBS mutations by WGS in tumor tissue (unfiltered, filtered): 4764, 1554
CGPLPA787
WGS data: Tumor (Yes), Normal (Yes), Plasma (10 timepoints)
Number of SBS mutations by WGS in tumor tissue (unfiltered, filtered): 18331, 13335
CGPLPA788
WGS data: Tumor (Yes), Normal (Yes), Plasma (4 timepoints)
Number of SBS mutations by WGS in tumor tissue (unfiltered, filtered): 5187, 1124
CGPLPA789
WGS data: Tumor (Yes), Normal (Yes), Plasma (5 timepoints)
Number of SBS mutations by WGS in tumor tissue (unfiltered, filtered): 5343, 2806
CGPLPA790
WGS data: Tumor (Yes), Normal (Yes), Plasma (4 timepoints)
Number of SBS mutations by WGS in tumor tissue (unfiltered, filtered): 15640, 5730
Radiation
Immunotherapy
CT scan
Last follow up
Sum of target lesions (mm)
WGMAF (%)
Targeted MAF (%)
ARTEMIS–DELFI score
CA 19–9 (U/mL)
RECIST 1.1
Baseline
PR
SD
PD
Survival at last follow up
Alive
Deceased

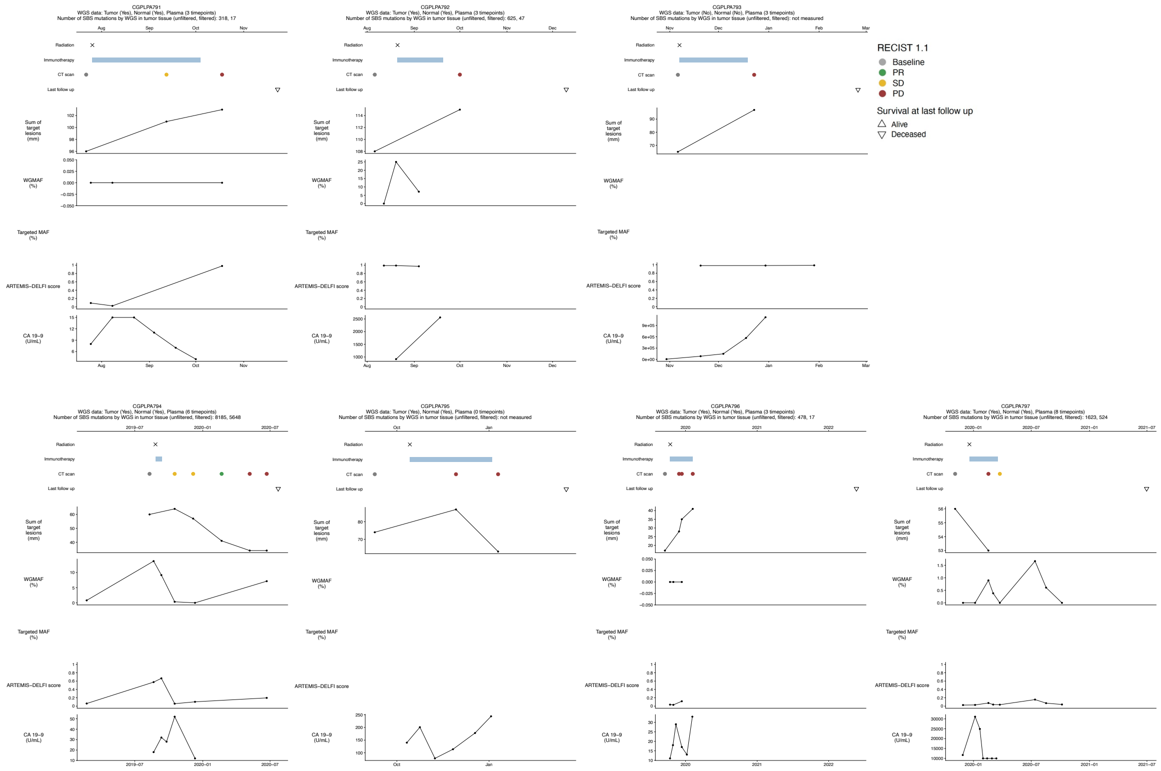

fig. S19. Longitudinal evaluation of patient response to treatment in CheckPAC trial using imaging and liquid biopsies. Response to treatment is shown for all patients. Patient clinical paths are shown in the top panels, followed by methodologies below for monitoring response to treatment. For the top panels, patient treatment is indicated in the top two rows, radiologic assessments are plotted in the third row and last follow-up is indicated in the fourth row. Sum of target lesions were assessed from standard-of-care CT scans. Available WGMAF, targeted MAF, and ARTEMIS-DELFI scores are plotted for each of the blood draw timepoints for each patient. CA19-9 concentrations were measured at regular intervals throughout treatment.

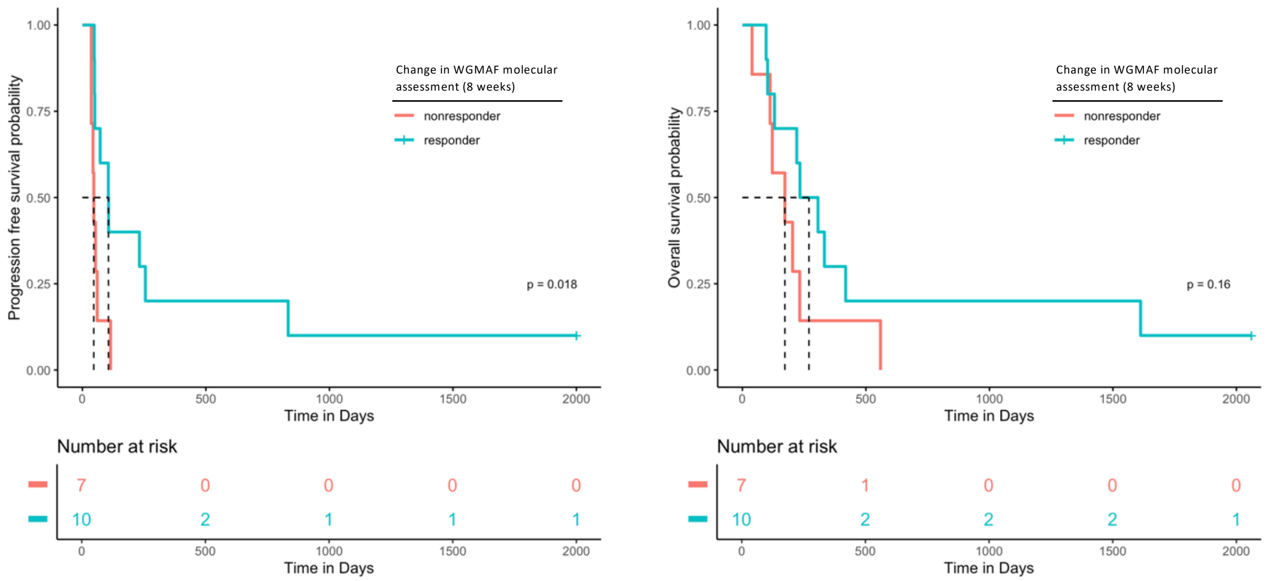

fig. S20. Change in WGMAF values stratify progression-free at 8-week timepoint in CheckPAC trial. Kaplan-Meier curves of progression-free survival probability and overall survival probability based on increase or decrease in WGMAF at 8-week timepoint.

A

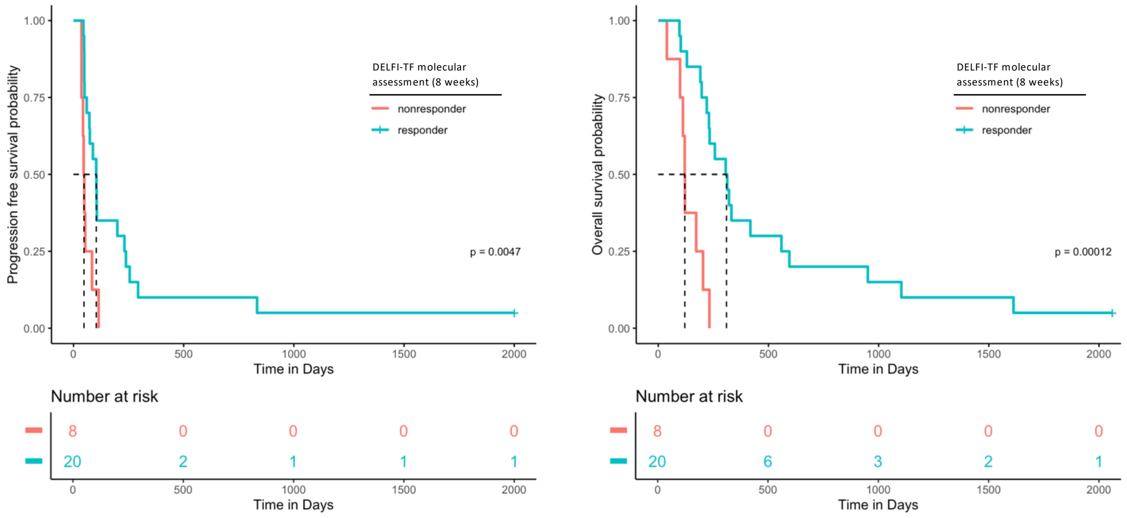

B

Subgroup	Patients	Hazard Ratio (95% CI)	P value
**Age, years**			
> 65	15	1	
≤ 65	13	0.71 (0.30-1.67)	0.44
**ECOG PS**			
0	18	1	
1	10	1.63 (0.60-4.42)	0.34
**Weight loss, %**			
< 5	14	1	
≥ 5	14	0.73 (0.26-2.11)	0.56
**MGPS**			
0	11	1	
1	14	0.76 (0.30-1.92)	0.56
2	3	1.46 (0.35-6.11)	0.61
**Treatment**			
RT+Nivo	10	1	
RT+Nivo/Ipi	18	0.67 (0.23-1.98)	0.47
**DELFI TF**			
Non-responder	8	1	
Responder	20	0.14 (0.04-0.49)	0.002

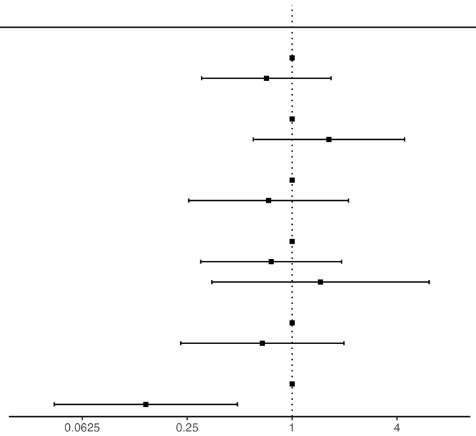

fig. S21. Changes in DELFI-TF scores between baseline and 8-week time point scores are predictive of progression-free and overall survival in CheckPAC trial. (A) Kaplan-Meier curves of progression-free survival probability and overall survival probability based on increase or decrease in DELFI-TF score at 8-week timepoint. (B) Multivariate cox proportional hazard analyses were generated for DELFI-TF and fit to overall survival adjusting for clinical subgroups. Subgroups previously shown to be significant in univariate analyses (*7*) were included in the multivariate analysis. Hazard ratios and P values are indicated on the right for individuals in the CheckPAC study.

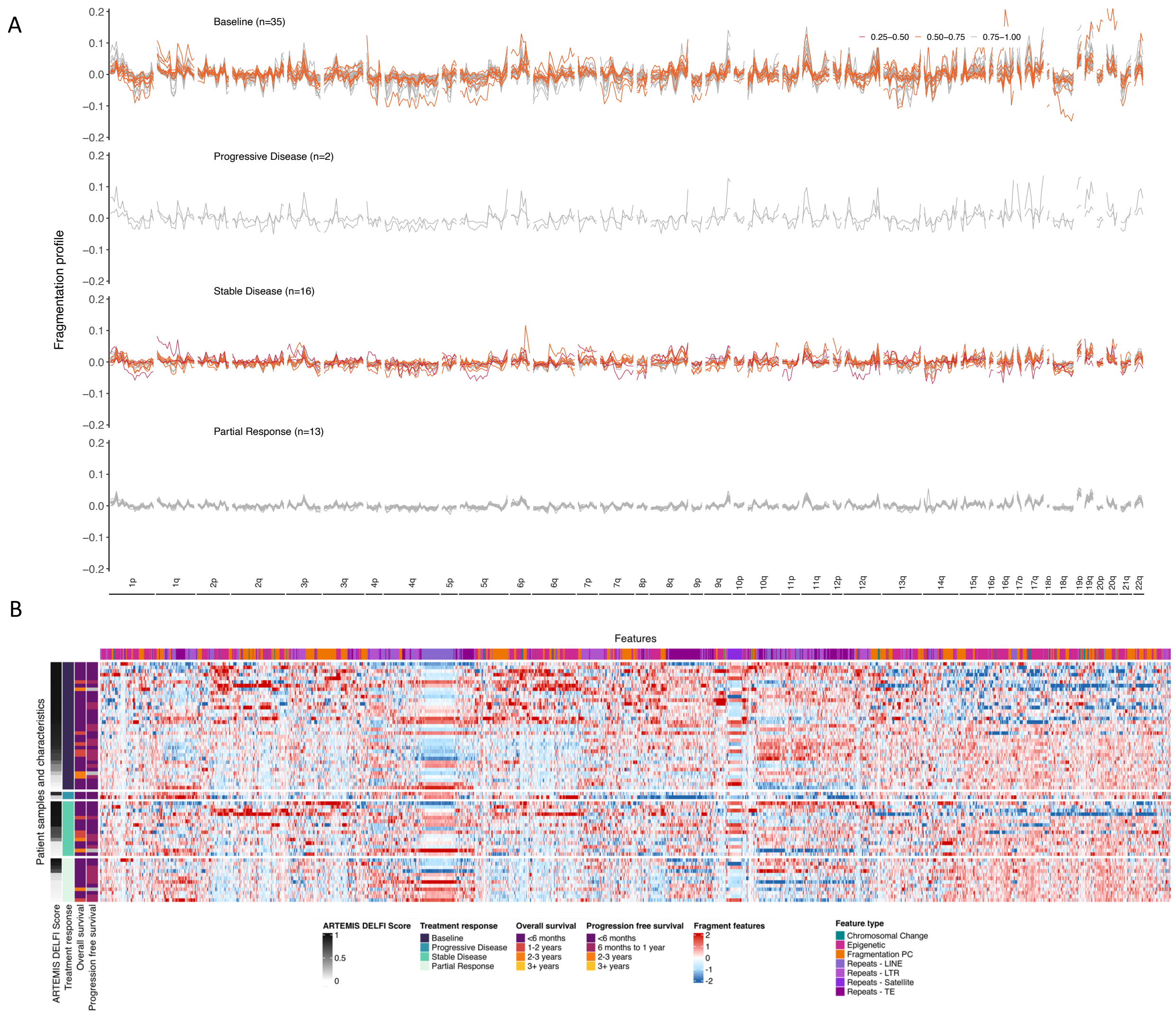

fig. S22. DELFI features correlate with clinical response of patients in PACTO cohort. (A) cfDNA fragmentation profiles from CheckPAC patients are shown as short (100 - 150 bp) to long (151 - 220 bp) ratios of fragment sizes in 473 bins 5-Mb in size across the genome. Profiles are shown for all patients with plasma samples at baseline, and at follow-up for each of the clinical RECIST 1.1 response categories. Each profile is colored by correlation to the median of 10 healthy reference samples. (B) The vertical axis is categorized by all patients with plasma samples at baseline, and at follow-up for each of the clinical RECIST 1.1 response categories and sorted by ARTEMIS-DELFI scores in descending order. All molecular features evaluated are plotted along the horizontal axis and colored by feature type. The heatmap color scale reflects the deviation of cfDNA features as compared to the mean of 10 non-cancer individuals.

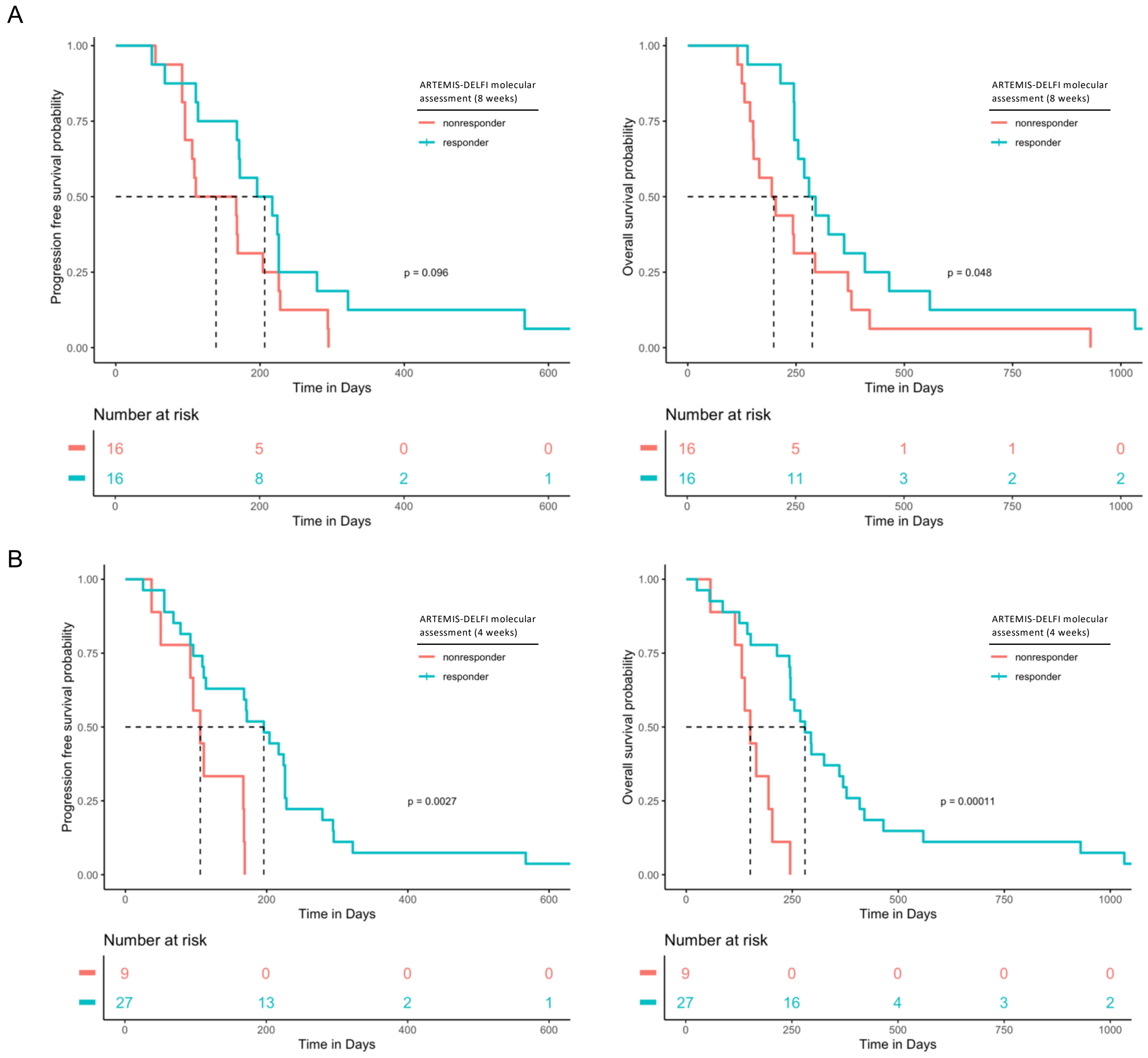

fig. S23. ARTEMIS-DELFI scores predict survival for patients in PACTO trial. (**A**) Kaplan-Meier curves of progression-free survival probability and overall survival probability based on median landmark ARTEMIS-DELFI score at 8 weeks. Patients are classified as responders or non-responders if follow-up ARTEMIS-DELFI scores are below or above the median follow-up score, respectively. (**B**) Kaplan-Meier curves of progression-free survival probability and overall survival probability based on fast-fail landmark ARTEMIS-DELFI score after one cycle of treatment. Patients are classified as responders or non-responders if follow-up ARTEMIS-DELFI scores are below or above the median follow-up score, respectively.

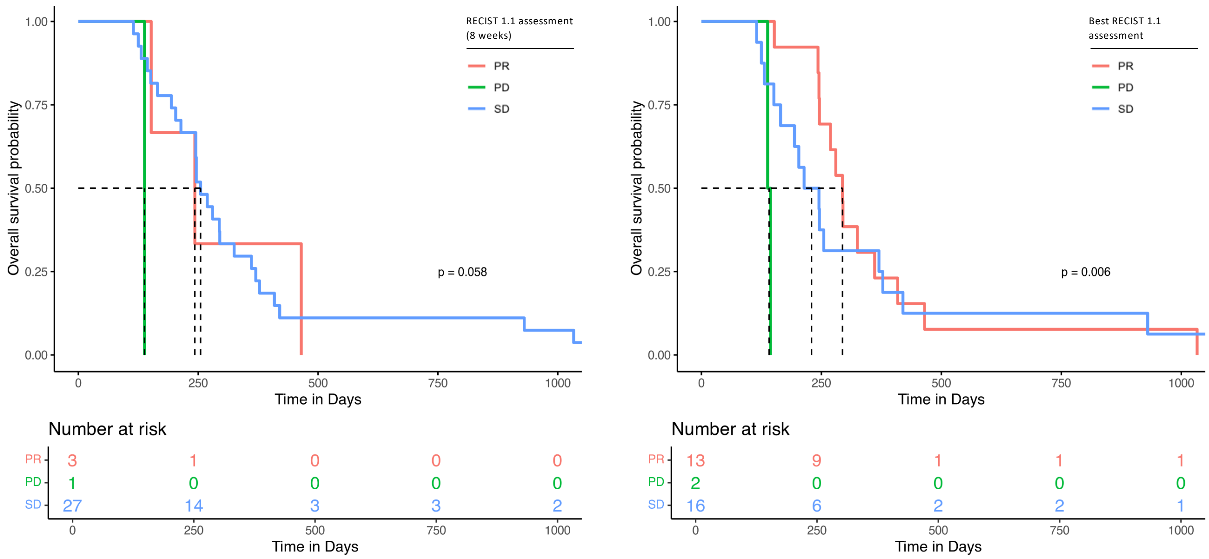

fig. S24. Survival analyses for RECIST scoring at first follow-up scan and for BOR RECIST in the PACTO trial. Overall survival probability is shown for each of the response categories for RECIST 1.1 score at the first follow-up at 8-weeks on the left, and for best overall response by RECIST 1.1 on the right.

List of Supplementary Tables

Supplementary Table S1. Clinical information of CheckPAC patients analyzed

Supplementary Table S2. Clinical information of PACTO patients analyzed

Supplementary Table S3. Summary of tumor and matched normal samples from CheckPAC patients

Supplementary Table S4. Mutational landscape of pancreatic cancers from CheckPAC patients

Supplementary Table S5. Summary of whole genome cfDNA analyses of CheckPAC patients

Supplementary Table S6. Summary of whole genome cfDNA analyses of PACTO patients
